# EpCAM-dependent extracellular vesicles from intestinal epithelial cells maintain intestinal tract immune balance

**DOI:** 10.1038/ncomms13045

**Published:** 2016-10-10

**Authors:** Lingling Jiang, Yingying Shen, Danfeng Guo, Diya Yang, Jiajun Liu, Xuefeng Fei, Yunshan Yang, Buyi Zhang, Zhendong Lin, Fei Yang, Xiaojian Wang, Keyi Wang, Jianli Wang, Zhijian Cai

**Affiliations:** 1 Institute of Immunology, Zhejiang University School of Medicine, Hangzhou 310058, China; 2 Department of Chemotherapy, Zhejiang Cancer Hospital, Hangzhou 310022, China; 3 Department of Pathology, Second Affiliated Hospital, Zhejiang University School of Medicine, Hangzhou 310009, China; 4 Department of Gynecology and Obstetrics, Second Affiliated Hospital, Wenzhou Medical University, Wenzhou 325035, China; 5 Department of Nutrition and Food Hygiene, Zhejiang University School of Public Health, Hangzhou 310058, China; 6 Chronic Disease Research Institute, Zhejiang University School of Public Health, Hangzhou 310058, China; 7 Central Laboratory, Nanjing Medical University, Affiliated Hangzhou Hospital (Hangzhou First People's Hospital), Hangzhou 310006, China

## Abstract

How the intestinal tract develops a tolerance to foreign antigens is still largely unknown. Here we report that extracellular vesicles (EVs) with TGF-β1-dependent immunosuppressive activity are produced by intestinal epithelial cells (IECs) under physiological conditions. Transfer of these EVs into inflammatory bowel disease (IBD) mice induced by dextran sulfate sodium salt decreases IBD severity by inducing regulatory T cells and immunosuppressive dendritic cells. In contrast, decreased endogenous EV production promotes IBD development. IECs produce EVs with increased levels of TGF-β1 upon IBD development in an ERK-dependent manner. Furthermore, these EVs tend to localize in the intestinal tract associated with epithelial cell adhesion molecule (EpCAM). Knockdown of EpCAM *in vivo* increases the severity of murine IBD, and the protective effect of EVs from IECs with decreased EpCAM on murine IBD is blunted. Therefore, our study indicates that EVs from IECs participate in maintaining the intestinal tract immune balance.

## Abstract (web summary)

The intestinal tract is continually exposed to foreign material and gut homeostasis is dependent on tolerance. Here, the authors show that extracellular vesicles released from intestinal epithelial cells stimulate T regulatory cells and immunosuppressive dendritic cells.

## Graphical abstract

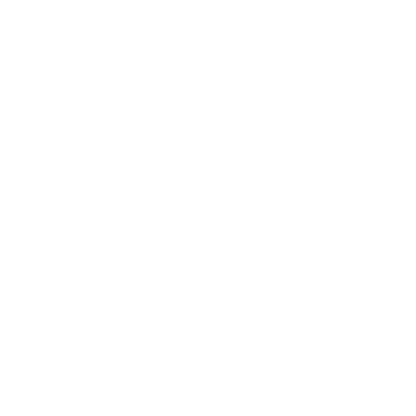

Immunotolerance is a specific condition in which the immune system shows an unresponsiveness or hyporesponsiveness to foreign harmless antigens or self-antigens^1^. Many mechanisms have been proposed, by which immunotolerance is maintained through regulation of activated T cells. These include T-cell anergy, regulatory T cells (Tregs) producing immunosuppressive cytokines and activation-induced T-cell apoptosis from undefined sites^2^. Because of their specific physiological feature, immunotolerance needs to be well established in some of human organs including the gut.

Approximately 30 kg of food proteins reach the human intestine in 1 year, and 130–190 g of these proteins are absorbed in the gut daily^3^. The ingestion of dietary antigens does not result in problematic immune reactions because of the effective creation of an immunotolerant environment in the gut. The mechanisms by which the intestinal tract achieves immunotolerance are under intensive study. It has been revealed that a complex interplay of factors are involved in maintaining this environment, including the participation of Tregs, dendritic cells (DCs), CD8^+^ T cells, γδ T cells, regulatory B cells, IgA, commensal bacteria and massive cytokines, such as transforming growth factor (TGF)-β1 and interleukin (IL-10; refs 4, 5, 6, 7, 8). Although information on immunotolerance in the intestinal tract has been accumulating, there is still much that needs to be elucidated.

The breakdown of intestinal immunotolerance can result in autoimmune diseases of the gut such as inflammatory bowel disease (IBD). IBD, including Crohn’s disease and ulcerative colitis, is characterized by a chronic and exacerbated inflammation of the intestinal mucosa^9^. Many patients suffer from IBD because of the recurrent attacks characteristic of this disease. An understanding of the mechanism of intestinal immunotolerance is thus required for the development of new effective curative strategies for IBD.

Extracellular vesicles (EVs) with lipid bilayer structures have been the subject of increased focus as mediators for communication between cells^10^. EVs consist of apoptotic bodies, ectosomes, microparticles, microvesicles and exosomes. Apoptotic bodies are released when plasma membrane blebbing occurs during apoptosis. Ectosomes, microparticles and microvesicles are 100–1,000 nm vesicles released by budding from the plasma membrane^11^,^12^. Exosomes are 30–150 nm vesicles released by the fusion of multivesicular bodies with the plasma membrane^12^. EVs have been shown to generate pleiotropic effects on the immune system including immune activation and suppression^13^,^14^. Immunosuppressive EVs exist under both physiological and pathological conditions. For example, Fas ligand-positive EVs released from human placenta have been demonstrated to inhibit T-cell signalling^15^. Exosome-like particles released from thymic cells can induce the development of Foxp3^+^ Tregs^16^, and EVs produced by synovial fibroblasts of individuals with rheumatoid arthritis delayed activation-induced cell death^17^. Interestingly, MHC-II^+^A33^+^ EVs produced by intestinal epithelial cells (IECs)^18^, and exosome-like ‘tolerosomes’, were found in the rat serum after feeding antigens^19^. These results suggest that immunosuppressive EVs are likely released from the intestine; however, the physiological characteristics of intestinal EVs and their function in maintaining intestinal immunotolerance are still unknown.

In this study, we find that EVs with high level of TGF-β1 are produced by IECs. Transfer of these EVs into dextran sulfate sodium salt (DSS)-induced IBD mice prevents the development of IBD by inducing Tregs and immunosuppressive DCs. However, inhibition of EV production *in vivo* exacerbates murine IBD. IECs of IBD mice produce EVs with increased levels of TGF-β1 by activating ERK. In addition, EVs tend to localize in the intestinal tract associated with epithelial cell adhesion molecule (EpCAM). Inhibition of EpCAM expression in colon aggravates murine IBD and the protective effect of EVs from IECs with decreased EpCAM on murine IBD is impaired. Therefore, our results reveal a still unknown mechanism for maintenance of intestinal immune balance, which is mediated by EVs from IECs.

## Results

### Intestine produces EVs containing high levels of TGF-β1

**Figure 1:**
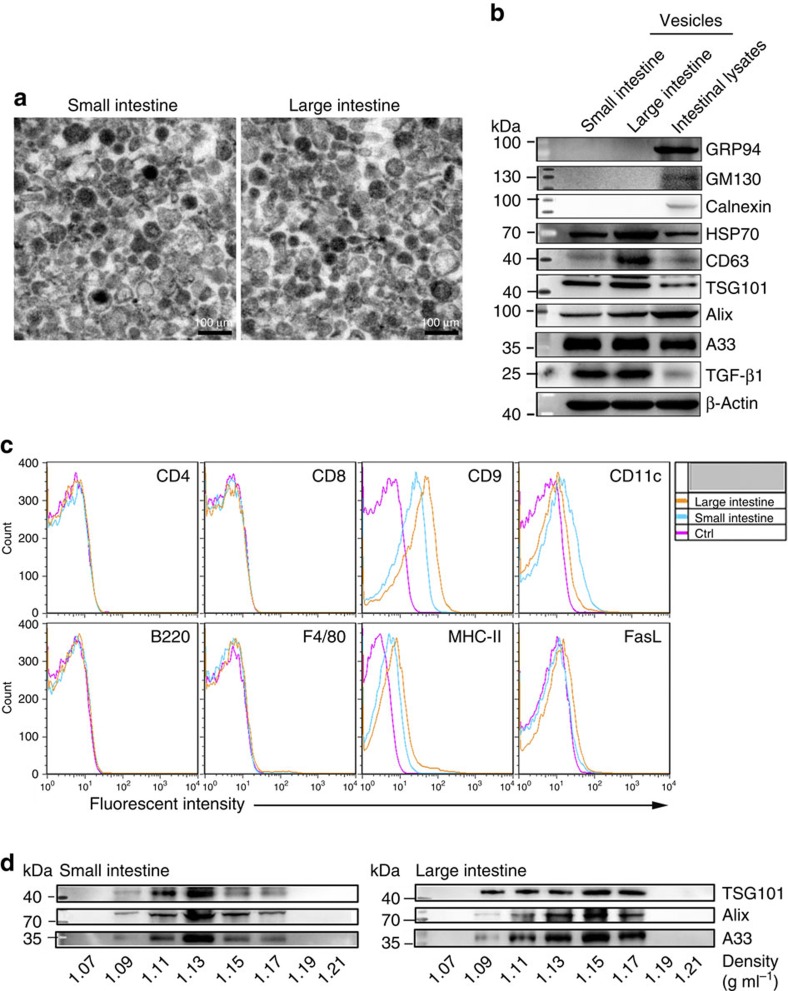
Characterization of intestinal EVs. Vesicles were isolated from the intestine according to a standard EV isolation procedure. (**a**) Representative electron micrograph of vesicles from small and large intestines. (**b**) A total of 20 μg of vesicles and total intestinal lysates were lysed and immunoblotted with corresponding antibodies. (**c**) After being absorbed on latex beads, the phenotype of vesicles from small and large intestine was analysed using FACS with the indicated antibodies. (**d**) A total of 100 μg of isolated vesicles from small or large intestines were loaded on a continuous sucrose gradient, and the fractions were analysed by western blot using the indicated antibodies. Data are representative of three independent experiments.

The EVs were isolated by ultracentrifugation after grinding and enzymatic digestion of intestinal tissues. Vesicles from small and large intestines ranged in size from 50 to 100 nm (Fig. 1a). Size distribution analysis revealed that the mean size of the vesicles from the small and large intestines was 75.76 and 73.27 nm with Ζ-potential of −22.22 and −22.33 mV, respectively (Supplementary Table 1). Both vesicles were positive for HSP70, CD63, TSG101, Alix and CD9 molecules, but are negative for endoplasmic reticulum-residing protein GRP94, Calnexin and Golgi apparatus-residing protein GM130 (Fig. 1b,c). In addition, both vesicles were positive for MHC-II, A33 (IEC-specific marker) and CD11c (DC-specific marker) and negative for CD4, CD8, B220 and F4/80 (Fig. 1b,c), suggesting the derivation of these vesicles from IECs and DCs. Interestingly, both vesicles were rich in TGF-β1, and vesicles from the large, but not the small intestine contained low amounts of FasL (Fig. 1c). To further characterize the vesicles, we floated them on a sucrose gradient, and found that A33, TSG101 and Alix molecules were enriched in the density of 1.09–1.17 g ml^−1^ (Fig. 1d). To exclude the possibility that the EVs were mixed with large amounts of intracellular vesicles or cell debris, intestinal tissues were mechanically homogenized, which would lead to the release of intracellular vesicles and production of numbers of cell debris. We obtained 2.6 times more vesicles by this method (Supplementary Fig. 1a). GRP94 and Calnexin, absent in EVs, were detected in these vesicles (Supplementary Fig. 1b). Altogether, these results demonstrate that the TGF-β1-containing EVs were released from the intestine under physiological conditions.

### IEC-secreted EVs inhibit CD4^+^ T-cell proliferation *in vitro*

**Figure 2:**
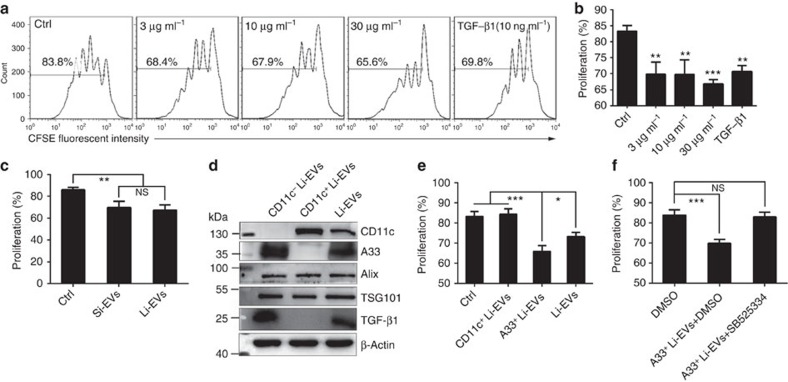
Li-EVs from IECs inhibit CD4^+^ T-cell proliferation *in vitro*. (**a**) Overall, 1 × 10^6 ^ml^−1^ carboxyfluorescein succinimidyl ester (CFSE)-labelled CD4^+^ T cells were seeded into 96-well plate and proliferation of T cells was elicited by 1 μl anti-CD3/CD28-coated beads. The indicated concentration of Li-EVs was added. TGF-β1 (10 ng ml^−1^) was added as a positive control. After culture for 3 days, proliferation of CD4^+^ T cells was analysed using FACS. (**b**) The data in **a** were statistically analysed (*n*=9). (**c**) The proliferation assay described in **a** was performed in the presence of 30 μg ml^−1^ Si-EVs or Li-EVs. The proliferation of CD4^+^ T cells was statistically analysed (*n*=9). (**d**) CD11c^+^ and CD11c^−^ Li-EVs were isolated by CD11c^+^ magnetic beads. CD11c, A33, Alix, TSG101, TGF-β1 and β-Actin in CD11c^+^, CD11c^−^ and total Li-EVs were detected by western blot analysis. (**e**) The proliferation assay described in **a** was performed in the presence of 30 μg ml^−1^ CD11c^+^, A33^+^ and total Li-EVs. The proliferation of CD4^+^ T cells was statistically analysed (*n*=9). (**f**) The proliferation assay described in **a** was performed in the presence of 30 μg ml^−1^ A33^+^ Li-EVs with or without 0.5 μg ml^−1^ TGF-β1 signalling inhibitor (SB525334). The proliferation of CD4^+^ T cells was statistically analysed (*n*=9). Data are representative of three independent experiments or are shown as mean values±s.e.m. pooled from three independent experiments. *P* values were generated by one-way analysis of variance (ANOVA), followed by Newman–Keuls multiple comparison test using GraphPad Prism 5, versus Ctrl in **a** (*P<0.05, **P<0.01, ***P<0.001, NS, not significant).

Because EVs from the intestine contained high levels of TGF-β1, we examined the effect of these EVs on CD4^+^ T-cell proliferation *in vitro*. As expected, EVs from the large intestine (Li-EVs) inhibited the CD4^+^ T-cell proliferation in a dose-dependent manner (Fig. 2a,b). Since there was no statistical difference in the inhibitory ability between EVs from the small intestine (Si-EVs) and Li-EVs (Fig. 2c), Li-EVs were used in the subsequent experiments. Because of the low amount of FasL, we examined whether Li-EVs could inhibit the CD4^+^ T-cell proliferation through inducing apoptosis and found that Li-EVs did not affect CD4^+^ T-cell apoptosis (Supplementary Fig. 2a,b).

From the preceding results, Li-EVs may be released from DCs and IECs. To determine which subset of Li-EVs was responsible for the suppressive effect, we isolated CD11c^+^ and CD11c^−^ Li-EVs using CD11c^+^ magnetic beads. As shown in Fig. 2d, CD11c^+^ Li-EVs were positive for CD11c and negative for A33, but CD11c^−^ Li-EVs were positive for A33 and negative for CD11c. Both types of isolated EVs were positive for TSG101 and Alix. In addition, TGF-β1 was positive in CD11c^−^ Li-EVs, but negative in CD11c^+^ Li-EVs (Fig. 2d). Size distribution analysis revealed that the mean size of CD11c^−^ Li-EVs and CD11c^+^ Li-EVs was 71.83 and 80.90 nm with Ζ-potential of −20.70 and −19.43 mV, respectively (Supplementary Table 1). These results indicated a successful isolation of CD11c^+^ Li-EVs and A33^+^ Li-EVs. Then, we examined the effect of these two subsets of EVs on CD4^+^ T-cell proliferation. At a concentration of 30 μg ml^−1^, only A33^+^ Li-EVs but not CD11c^+^ Li-EVs inhibited the CD4^+^ T-cell proliferation. A33^+^ Li-EVs were more immunosuppressive than total Li-EVs at the same concentration (Fig. 2e). This suggests that Li-EVs from IECs are immunosuppressive.

To elucidate the role of TGF-β1 in the immunosuppressive potential of A33^+^ Li-EVs, we pretreated the CD4^+^ T cells with SB525334, a potent and selective inhibitor of TGF-β receptor I. After blocking TGF-β1 signalling, A33^+^ Li-EVs hardly inhibited CD4^+^ T-cell proliferation (Fig. 2f). In addition, the inhibitory effect of A33^+^ Li-EVs on CD4^+^ T-cell proliferation was totally abolished by anti-TGF-β1-neutralized antibodies (Supplementary Fig. 2c). To further confirm this, we isolated CD4^+^ T cells from *Smad3+/−* mice, which are deficient in TGF-β1 signalling, and performed the proliferation assay. As expected, A33^+^ Li-EVs did not affect CD4^+^ T-cell proliferation from *Smad3+/−* mice (Supplementary Fig. 2d). Taken together, these results indicate that Li-EVs from IECs can inhibit CD4^+^ T-cell proliferation by TGF-β1 *in vitro*.

### A33^+^ Li-EVs alleviate IBD through TGF-β1 signalling

**Figure 3:**
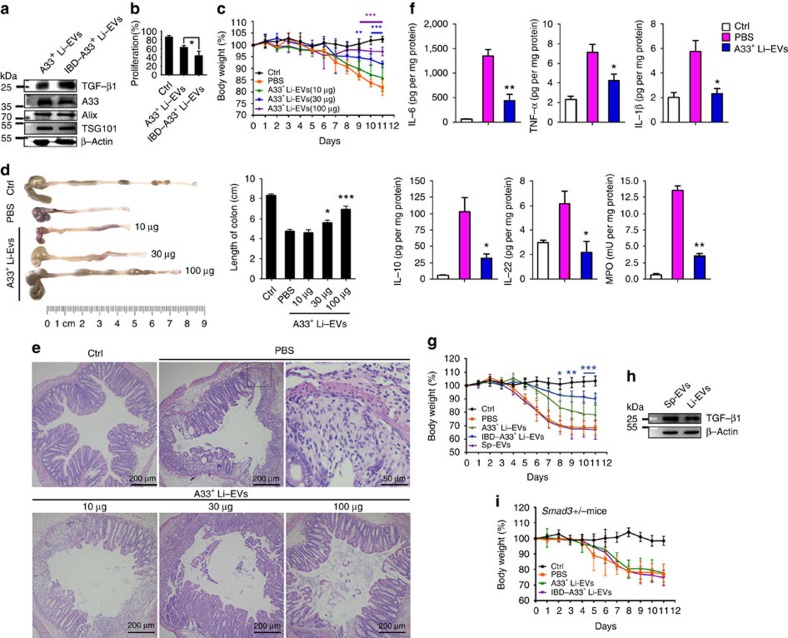
A33^+^ Li-EVs alleviate IBD through TGF-β1 signalling. (**a**) A33^+^ Li-EVs were isolated from healthy control or 2% DSS-induced IBD mice. TGF-β1, A33 and β-Actin were detected by western blot analysis. (**b**) The CD4^+^ T-cell proliferation assay was performed as described above in the presence of 30 μg ml^−1^ Ctrl or IBD-A33^+^ Li-EVs, and the results were statistically analysed (*n*=9). (**c**–**f**) Mice were fed with drinking water containing 2% DSS on day 0. On days −2 and 2, the mice were intravenously treated with the indicated dose of A33^+^ Li-EVs. The body weights were measured daily (**c**). Appearance (left) and statistical analysis (right) of colonic length on days 11 (**d**). Histological appearance on day 11. Representative colonic sections stained with haematoxylin and eosine (H&E). In the PBS group, the right image is a magnified region of the left image (**e**). IL-1β, IL-6, TNF-α, IL-10, IL-22 levels and MPO activity in colon tissue were measured on day 11 (**f**). (**g**) IBD mice were treated with 100 μg A33^+^ Li-EVs, IBD-A33^+^ Li-EVs and Sp-EVs on days −2 and 2. The body weights were measured daily. (**h**) TGF-β1 in A33^+^ Li-EVs and Sp-EVs was detected using western blot analysis. (**i**) IBD was introduced into *Smad3+/−* mice and treated with 100 μg A33^+^ Li-EVs and IBD-A33^+^ Li-EVs on days −2 and 2. The body weights were measured daily. Ctrl group, mice received normal drinking water; PBS group, mice with drinking water containing 2% DSS and intravenously treated with PBS on days −2 and 2. (**a**,**b**,**d**–**f**) Data are representative of three independent experiments or shown as mean values±s.e.m. pooled from three independent experiments; (**c**,**g**,**i**) data are presented as the mean±s.d. from one of the three independent experiments (*n*=5 per group). *P* values were generated by one-way ANOVA in **b**,**d**,**f**, or two-way ANOVA in **c**,**g**,**i**, followed by Newman–Keuls multiple comparison test using GraphPad Prism 5 (*P<0.05, **P<0.01, ***P<0.001), corresponding colour indicating the relevant group versus PBS group in **c**, versus PBS group in **d**,**f**; IBD-A33^+^ Li-EVs versus A33^+^ Li-EVs in **g**.

The A33^+^ Li-EVs’ inhibitory effect on CD4^+^ T-cell proliferation *in vitro* suggested that A33^+^ Li-EVs may be implicated in maintaining intestinal immunotolerance. Although A33^+^ Li-EVs and A33^+^ Li-EVs of IBD mice (IBD-A33^+^ Li-EVs) had similar size distributions (Supplementary Table 1), IBD-A33^+^ Li-EVs contained more TGF-β1 as measured by western blot analysis, and showed a stronger inhibitory effect on CD4^+^ T-cell proliferation *in vitro* than did A33^+^ Li-EVs (Fig. 3a,b). The increased amounts of TGF-β1 in IBD-A33^+^ Li-EVs were also confirmed using ELISA (Supplementary Fig. 3a). In addition, we also found that the amounts of TGF-β1 in A33^+^ Li-EVs gradually increased with the development of IBD (Supplementary Fig. 3b). These results suggested that A33^+^ Li-EVs probably control immune balance in the intestine. If this is true, exogenous A33^+^ Li-EVs could decrease the severity of DSS-induced murine acute IBD. After intravenous transfer of A33^+^ Li-EVs, we observed markedly decreased body weight loss of IBD mice in a dose-dependent manner (Fig. 3c). DSS-induced shortening of colonic length was also decreased by A33^+^ Li-EVs in a dose-dependent manner (Fig. 3d). Histological assessment of colonic damage revealed a large number of leukocytes (mainly neutrophils and eosinophils) infiltrated into the mucosa and submucosa. There was also extensive damage of glandular structure in colon tissue of mice with drinking water containing 2% DSS, and intravenously treated with PBS, suggesting obvious acute inflammation (Fig. 3e). Mice treated with A33^+^ Li-EVs had less damage in colon tissue, showing more conserved glandular structure and limited leukocyte infiltrations (Fig. 3e). The production of IL-6, tumour necrosis factor (TNF)-α, IL-1β, IL-10 and IL-22 in colon tissue was significantly inhibited by A33^+^ Li-EVs (Fig. 3f). Myeloperoxidase (MPO) is a marker for neutrophil and helps in assessing the neutrophil influx into inflamed tissue^20^. MPO activity can be used as a marker of inflammation. MPO activity was also inhibited by A33^+^ Li-EVs (Fig. 3f).

IBD is a chronic inflammatory disorder. Therefore, we sought to examine the effect of A33^+^ Li-EVs on the severity of colitis in a model of chronic IBD induced according to the protocol shown in Supplementary Fig. 4a. After A33^+^ Li-EV treatment, mice showed the trend of slower weight loss, but with no statistical difference (Supplementary Fig. 4b). The length of colons of A33^+^ Li-EV-treated mice was longer than that of PBS-treated mice (Supplementary Fig. 4c). Histological examination revealed substantial leukocyte (mainly lymphocytes and eosinophils) infiltration, fibroblast hyperplasia and angiogenesis, and an extensive damage of glandular structure in colon tissue of PBS-treated mice, indicating chronic inflammation (Supplementary Fig. 4d). There was less leukocyte infiltration, fibroblast hyperplasia and local damage of glandular structure in colon tissue of A33^+^ Li-EV-treated mice (Supplementary Fig. 4d). In addition, A33^+^ Li-EV treatment decreased production of IL-6, TNF-α, IL-1β, IL-10, IL-22 and activity of MPO (Supplementary Fig. 4e). Immunohistochemistry (IHC) demonstrated that A33^+^ Li-EV treatment decreased numbers of CD4^+^ and CD8^+^ T cells, neutrophils, macrophages and DCs (Supplementary Fig. 4f). Altogether, these results suggest that A33^+^ Li-EVs protected mice from acute and chronic IBD.

In accordance with *in vitro* results, A33^+^ Li-EVs in IBD mice were more effective in preventing DSS-induced acute weight loss compared with control mice (Fig. 3g). To determine whether the IBD-protection function was A33^+^ Li-EV-specific, we assessed the effects of EVs from the spleen (Sp-EVs) on acute IBD and found that Sp-EVs did little to prevent the development of acute IBD compared with A33^+^ Li-EVs (Fig. 3g). Contrary to previous publications, which reported that Sp-EVs contained no TGF-β1 (ref. 16), we detected higher levels of TGF-β1 in Sp-EVs (Fig. 3h). Therefore, we further confirmed the role of TGF-β1 in A33^+^ Li-EXO and IBD-A33^+^ Li-EXO. We examined their effects on the development of acute IBD in *Samd3+/−* mice. As shown in Fig. 3i, Li-EXO did not prevent DSS-induced weight loss in *Samd3+/−* mice. Together, these results indicate that A33^+^ Li-EVs prevented the development of murine IBD through TGF-β1 signalling, and higher levels of TGF-β1, but not other factors determined the enhanced efficacy of IBD-A33^+^ Li-EVs to prevent IBD development.

### A33^+^ Li-EVs alleviate IBD through inducing Tregs

**Figure 4:**
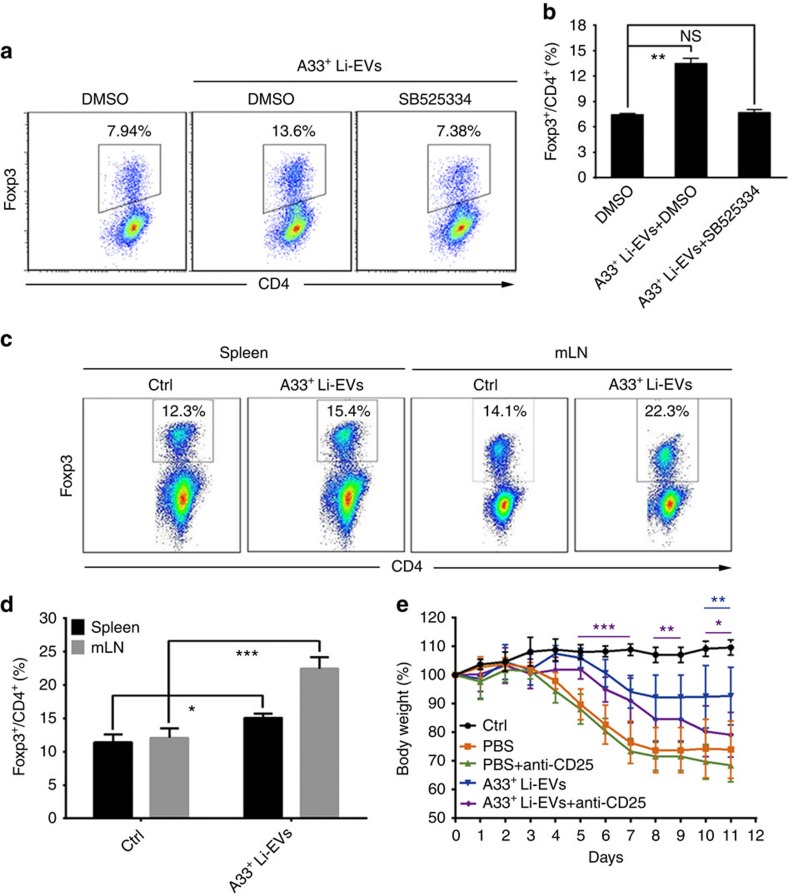
Effects of A33^+^ Li-EVs on Tregs and the severity of IBD. (**a**) In all, 1 × 10^6 ^ml^−1^ naive CD4^+^ T cells were seeded into a 96-well plate and stimulated with 1 μl anti-CD3/CD28-coated beads. Then, 30 μg ml^−1^ A33^+^ Li-EVs with or without 0.5 μg ml^−1^ SB525334 was added. After culture for 3 days, the differentiation of CD4^+^Foxp3^+^ Tregs was analysed using FACS. (**b**) The data in **a** were statistically analysed (*n*=9). (**c**) Mice received one intravenous injection of 100 μg A33^+^ Li-EVs and were killed, and lymphocytes from the spleen and mLNs were isolated 3 days later. The percentage of CD4^+^Foxp3^+^ Tregs was analysed using FACS. (**d**) The data in **c** were statistically analysed (*n*=9). (**e**) IBD was introduced into the control or CD4^+^Foxp3^+^ Treg-depleted mice, and the mice were intravenously treated with 100 μg A33^+^ Li-EVs on days −2 and 2. The body weights of mice were measured daily. To deplete CD4^+^Foxp3^+^ Tregs, each mouse was intraperitoneally injected with 100 μg anti-mouse CD25 monoclonal antibodies or PBS as a control on days −8, −6 and −4. Ctrl group, mice that received normal drinking water; PBS group, mice with drinking water containing 2% DSS and intravenously treated with PBS on days −2 and 2. (**a**–**d**) Data are representative of three independent experiments or are shown as mean values±s.e.m. pooled from three independent experiments; (**e**) data are presented as the mean±s.d. from one of the three independent experiments (*n*=5 per group). *P* values were generated by one-way ANOVA in **b**,**d**, or two-way ANOVA in **e**, followed by Newman–Keuls multiple comparison test using GraphPad Prism 5 (*P<0.05, **P<0.01, ***P<0.001), purple or blue colour indicating PBS+anti-CD25 versus A33^+^ Li-EVs+anti-CD25 or A33^+^ Li-EVs versus A33^+^ Li-EVs+anti-CD25, respectively, in **e**.

We have previously reported that TGF-β1-containing EVs can induce Tregs to decrease DSS-induced IBD in mice^21^. We found that A33^+^ Li-EVs induced differentiation of CD4^+^Foxp3^+^ Tregs *in vitro* through TGF-β1 (Fig. 4a,b). In addition, A33^+^ Li-EVs also increased the percentage of CD4^+^Foxp3^+^ Tregs in the murine spleen and mesenteric lymph nodes (mLNs) *in vivo*. The increased rate of CD4^+^Foxp3^+^ Tregs in mLNs was greater than that in the spleen (Fig. 4c,d). To determine the role of CD4^+^Foxp3^+^ Tregs in the A33^+^ Li-EV-mediated decrease in severity of murine IBD, CD4^+^Foxp3^+^ Tregs were depleted by the pretreatment of mice with anti-CD25 monoclonal antibodies. The protective effect of A33^+^ Li-EVs against DSS-induced weight loss somewhat decreased (Fig. 4e). Together, these results suggest that Tregs partially contributed to A33^+^ Li-EV-mediated decrease in severity of DSS-induced murine IBD.

### A33^+^ Li-EVs alleviate IBD by inhibiting DC activation

**Figure 5:**
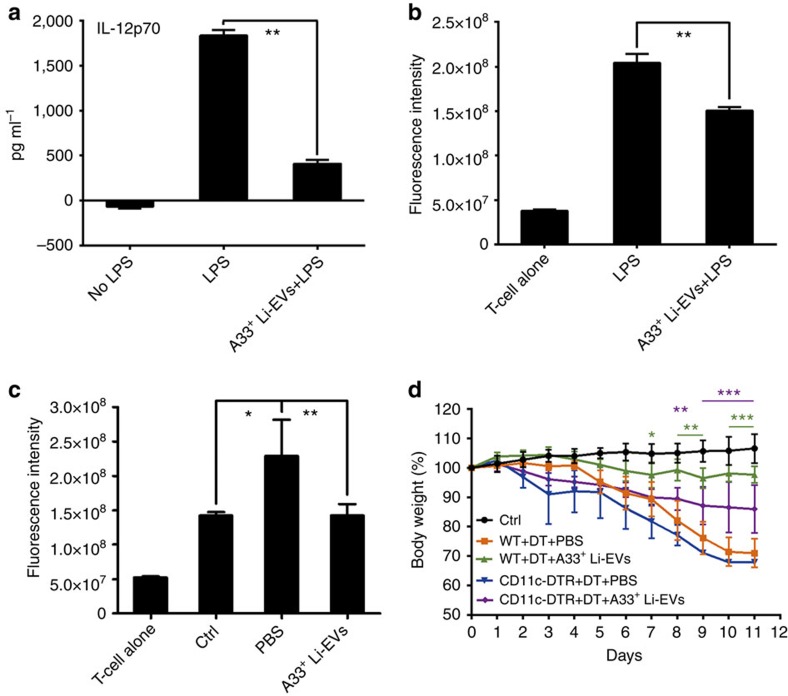
DCs are involved in alleviating IBD mediated by A33^+^ Li-EVs. BMDCs were stimulated with 1 μg ml^−1^ LPS or LPS plus 30 μg ml^−1^ A33^+^ Li-EVs for 24 h. (**a**) The level of IL-12p70 in the supernatant was measured using enzyme-linked immunosorbent assay (ELISA; *n*=9) and (**b**) BMDCs were collected and 1 × 10^5^ ml^−1^ BMDCs were cocultured with CD4^+^ T cells from BALB/c mice at the ratio of 1:10. The proliferation of CD4^+^ T cells was measured by alamar Blue assay (*n*=9). The value of fluorescent intensity as shown=(value of absolute fluorescent intensity−value of fluorescent intensity of DC alone). (**c**) DCs from healthy mice (Ctrl), PBS-treated IBD mice (PBS) and A33^+^ Li-EV-treated IBD mice (A33^+^ Li-EVs) were isolated on day 11, and the proliferation of CD4^+^ T cells was induced and measured as described in **b**. (**d**) CD11c-DTR and WT mice were fed with 2% DSS solution on day 0. Each mouse was intraperitoneally injected with DT 4 μg kg^−1^ body weight and intravenously injected with 100 μg A33^+^ Li-EVs on days −2 and 2. The body weight of mice was measured daily. Ctrl group, mice that received normal drinking water; PBS group, mice with drinking water containing 2% DSS and intravenously treated with PBS on days −2 and 2. (**a**–**c**) Data are shown as mean values±s.e.m. (*n*=9) pooled from three independent experiments; (**d**) data are presented as the mean±s.d. from one of the three independent experiments (*n*=5 per group). *P* values were generated by one-way ANOVA in **a**–**c**, or two-way ANOVA in **d**, followed by Newman–Keuls multiple comparison test using GraphPad Prism 5 (*P<0.05, **P<0.01, ***P<0.001), green or purple colour indicating WT+DT+A33^+^ Li-EVs versus CD11c-DTR+DT+A33^+^ Li-EVs or CD11c-DTR+DT+A33^+^ Li-EVs versus CD11c-DTR+DT+PBS in **d**.

Defects in DC function have been reported to contribute to the pathogenesis of IBD^22^. Thus, we determined whether A33^+^ Li-EVs could regulate DC function leading to the amelioration of IBD. We found that A33^+^ Li-EVs significantly inhibited lipopolysaccharide (LPS)-induced IL-12 secretion and antigen-presenting ability in the mixed lymphocyte reaction of DCs (Fig. 5a,b). To further confirm that A33^+^ Li-EVs could affect the DC function, we isolated DCs from PBS or A33^+^ Li-EV-treated IBD mice or healthy mice. When detected in mixed lymphocyte reaction, DCs from PBS-treated mice showed stronger antigen-presenting ability than did DCs from healthy mice (Fig. 5c). However, the antigen-presenting ability of DCs from A33^+^ Li-EV-treated mice was significantly decreased and is comparable to DCs from healthy mice (Fig. 5c). After intraperitoneal injection of diphtheria toxin (DT) in wild-type (WT) mice, A33^+^ Li-EVs still had a protective effect on DSS-induced IBD, suggesting that DT does not affect the development of DSS-induced IBD (Fig. 5d). The weight loss in mice injected with DT was significantly higher in CD11c-DTR mice than in WT mice after A33^+^ Li-EV treatment (Fig. 5d); however, there was still a significantly slower weight loss in DT-injected CD11c-DTR mice with A33^+^ Li-EV treatment when compared with DT-injected CD11c-DTR mice without A33^+^ Li-EV treatment (Fig. 5d). Together, these results indicate that A33^+^ Li-EVs alleviate the severity of DSS-induced IBD partially depending on DCs.

### Inhibition of endogenous EV production aggravates IBD

**Figure 6:**
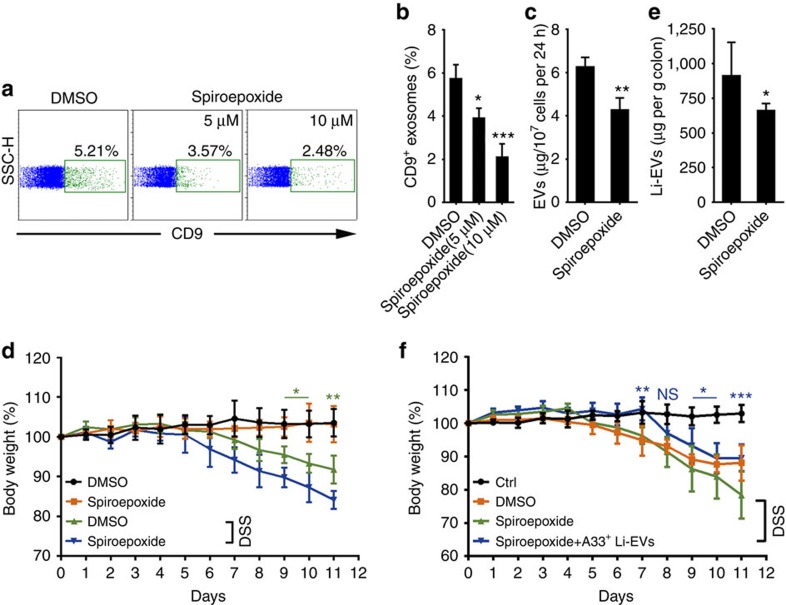
Inhibition of endogenic EV production and the IBD severity. (**a**) MC38 cells were treated with the indicated concentration of spiroepoxide for 24 h. The EVs in the supernatant of MC38 cells were absorbed by CD63-coated latex beads and the percentage of CD9-positive latex beads was detected using FACS. (**b**) The data in **a** were statistically analysed (*n*=9). (**c**) Overall, 1 × 10^7^ MC38 cells were treated with DMSO or 10 μM spiroepoxide for 24 h. The EVs in the culture supernatants were isolated, and the protein measured by a BCA assay and statistically analysed (*n*=9). (**d**) Mice were fed with water or 2% DSS solution on day 0 and received intraperitoneal injection of spiroepoxide (2 g kg^−1^ body weight) or DMSO control. The body weights of mice were measured daily. (**e**) The mice were killed on day 11 and Li-EVs were isolated and quantified. Quantity of the Li-EVs was normalized according to colon weight and statistically analysed (*n*=9). (**f**) Mice were treated as described in **d**. In addition to spiroepoxide injection, some mice were also intravenously injected with 100 μg A33^+^ Li-EVs. The body weight of mice was measured daily. Ctrl group, mice that received normal drinking water. (**a**–**c**,**f**) Data are representative of three independent experiments or are shown as mean values±s.e.m. pooled from three independent experiments; (**d**,**f**) data are presented as the mean±s.d. from one of the three independent experiments (*n*=5 per group). *P* values were generated by one-way ANOVA in **b**, or two-way ANOVA in **d**,**f**, followed by Newman–Keuls multiple comparison test, and by unpaired Student’s *t*-test in **c**,**e** using GraphPad Prism 5 (*P<0.05, **P<0.01, ***P<0.001), DMSO plus DSS versus Spiroepoxide plus DSS in **d**; Spiroepoxide+A33^+^ Li-EVs versus Spiroepoxide in **f**.

Because intestinal EVs are involved in maintaining intestinal tract immune balance, inhibition of the endogenous production of EVs probably makes the mice more susceptible to the DSS-induced IBD. Spiroepoxide has been reported to inhibit exosome release^23^,^24^. First, we confirmed the inhibitory effect of spiroepoxide on EV release from MC38 cells (a murine colon adenocarcinoma cell line) and found that spiroepoxide inhibited EV release from MC38 cells in a dose-dependent manner (Fig. 6a,b). In addition, by protein measurements, we found that spiroepoxide treatment resulted in a 32% decrease in EV production from 10^7^ MC38 cells per 24 h (Fig. 6c). We found no obvious differences in TGF-β1 levels in colon tissues *in vivo* without or with spiroepoxide treatment (Supplementary Fig. 5a). After treatment with spiroepoxide, the mice given plain water showed no change of body weight compared with mice without spiroepoxide treatment. However, the mice with DSS in their water had more weight loss when compared with their corresponding control group (Fig. 6d). The quantity of Li-EVs significantly decreased, confirming the inhibitory effect on the release of Li-EVs *in vivo* (Fig. 6e). Furthermore, exogenous transfer with A33^+^ Li-EVs abolished the effect of spiroepoxide (Fig. 6f). Furthermore, spiroepoxide or spiroepoxide plus A33^+^ Li-EV treatment did not affect TGF-β1 levels in colon tissues of IBD mice (Supplementary Fig. 5b). Together, these results suggest that inhibition of the EV production *in vivo* can increase the susceptibility of mice to DSS-induced IBD.

### ERK is involved in increasing TGF-β1 in IBD-A33^+^ Li-EVs

**Figure 7:**
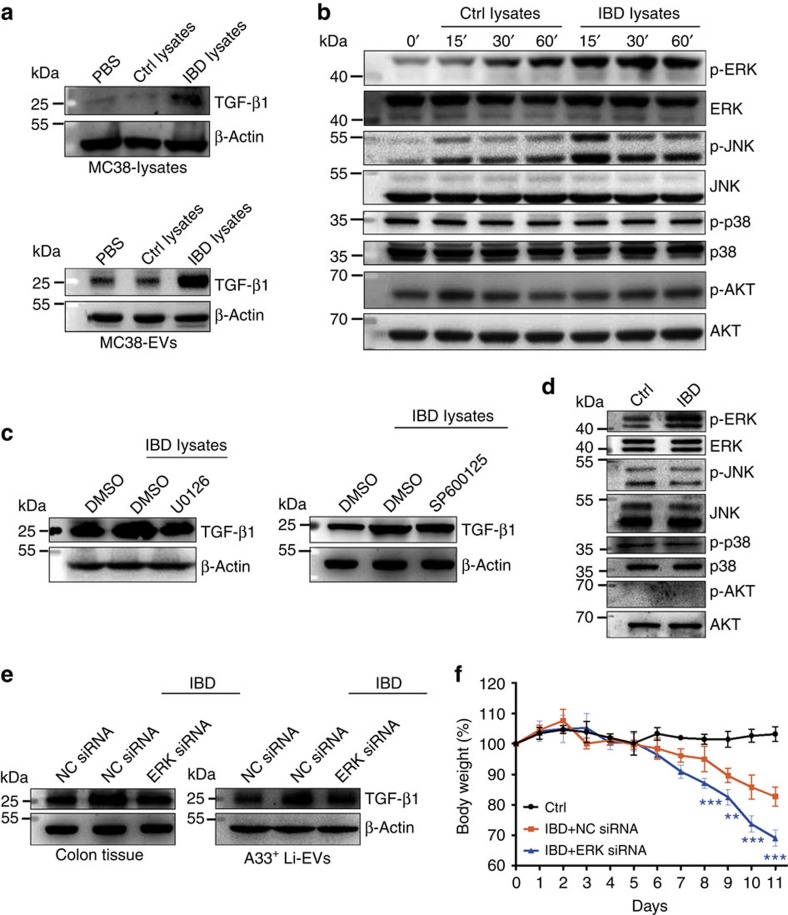
ERK and TGF-β1 levels in IBD-A33^+^ Li-EVs. (**a**) MC38 cells were treated with 1 mg ml^−1^ IBD lysates or Ctrl lysates for 24 h. TGF-β1 in MC38 cells or MC38-EVs was measured using western blot analysis. (**b**) MC38 cells were treated with 1 mg ml^−1^ IBD lysates or Ctrl lysates for the indicated times. Activation of ERK, JNK, p38 and ATK in MC38 cells was measured using western blot analysis. (**c**) MC38 cells were pretreated with DMSO, 10 μM ERK-specific inhibitor U0126 or JNK-specific inhibitor SP600125 for 30 min and then treated with 1 mg ml^−1^ IBD lysates or Ctrl lysates for 24 h. TGF-β1 in MC38-EVs was measured by western blot analysis. (**d**) Activation of ERK, JNK, p38 and ATK in colon tissues from healthy control or IBD mice was measured using western blot analysis. (**e**,**f**) Mice were fed with 2% DSS solution on day 0. Mice received intrarectal injection with 20 μg cholesterol-conjugated ERK or NC siRNA every other day on days 0–11. Twenty-four hours after the last injection, mice were killed. TGF-β1 in colon tissues and A33^+^ Li-EVs was measured using western blot analysis (**e**). The body weights of mice were measured daily (**f**). Ctrl group, mice that received normal drinking water. (**a**–**e**) Data are representative of three independent experiments; (**f**) data are presented as the mean±s.d. from one of the two independent experiments (*n*=5 per group). *P* values were generated by two-way ANOVA, followed by Newman–Keuls multiple comparison test using GraphPad Prism 5 (**P<0.01, ***P<0.001), IBD+NC siRNA versus IBD+ERK siRNA.

Because IBD-A33^+^ Li-EVs had higher levels of TGF-β1 than did A33^+^ Li-EVs, we measured TGF-β1 levels in colon tissues, and found higher TGF-β1 levels in colon tissues from IBD mice compared with healthy mice (Supplementary Fig. 6a). This suggested that the higher levels of TGF-β1 in parental cells might have resulted in the increased levels of TGF-β1 in IBD-A33^+^ Li-EVs, and not selective enrichment of TGF-β1 in EVs. After treatment with lysates from IBD (IBD lysates), but not with healthy control (Ctrl lysates) mice, MC38 cells, a colon tumour cell line derived from epithelial cells, showed increased expression of TGF-β1 (Fig. 7a). Similar to their parental cells, EVs from IBD lysate, but not Ctrl-lysate-treated MC38 cells contained higher levels of TGF-β1 (Fig. 7a).

ERK and AKT signalling pathways have been reported to increase TGF-β1 expression^25^,^26^. After IBD-lysate stimulation in MC38 cells, we found that ERK and JNK were activated (Fig. 7b). After pretreatment with an ERK-specific inhibitor, U0126, but not a JNK-specific inhibitor, SP600125, IBD lysates induced increased levels of TGF-β1 in MC38 cells (Supplementary Fig. 6b) and in MC38 cell-derived EVs (MC38-EVs) were totally abolished (Fig. 7c). Consistent with the *in vitro* findings in MC38 cells, we found an increase in ERK, without changes in JNK and p38 activation in colon tissues from IBD mice when compared with that from healthy control mice. In addition, phosphorylated AKT was barely detectable in the colon tissues from both IBD and healthy control mice (Fig. 7d). To knockdown ERK expression *in vivo*, we intrarectally injected cholesterol-conjugated ERK short interfering RNA (siRNA), and found that cholesterol-conjugated ERK siRNA but not negative control (NC) siRNA effectively decreased the levels of total ERK and phosphorylated ERK (p-ERK) in the large intestine (Supplementary Fig. 6c). TGF-β1 levels in colon tissues from healthy control mice and A33^+^ Li-EVs were also markedly decreased (Supplementary Fig. 6d). In addition, increased levels of TGF-β1 also obviously inhibited in colon tissues from IBD mice and IBD-A33^+^ Li-EVs after ERK knockdown (Fig. 7e). Mice injected with ERK siRNA showed more severe IBD than did mice injected with NC siRNA (Fig. 7f). Similar to the ERK knockdown results *in vivo*, intravenous injection of the ERK inhibitor CI-1040 prevented an increase in TGF-β1 in colon tissues from IBD mice and IBD-A33^+^ Li-EVs as well (Supplementary Fig. 6e). Furthermore, mice injected with CI-1040 showed more severe IBD than did mice injected with dimethylsulphoxide (DMSO; Supplementary Fig. 6f). To further confirm the function of ERK-induced increases in TGF-β1 in A33^+^ Li-EVs, we intravenously transferred A33^+^ Li-EVs from DMSO or CI-1040-treated IBD mice and found that A33^+^ Li-EVs from DMSO-treated IBD mice had less DSS-induced weight loss (Supplementary Fig. 6g) than did those from CI-1040-treated IBD mice. These results indicate that ERK is activated in IBD, and involves the upregulation of TGF-β1 expression in IECs, and subsequent release of EVs containing increased levels of TGF-β1.

### Higher TGF-β1 and p-ERK in IBD-patient intestinal tissues

**Figure 8:**
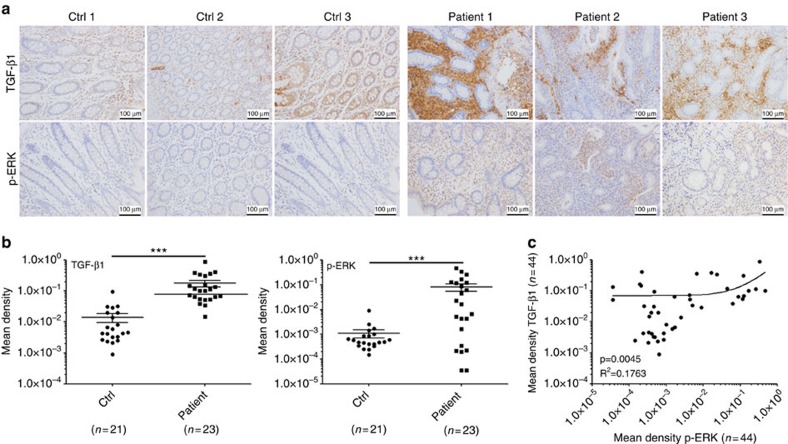
TGF-β1 and p-ERK levels in intestine tissues of IBD patients. (**a**) TGF-β1 and p-ERK expression in intestinal tissues from IBD patients or healthy controls were detected using IHC. Data are three representative images from the results of IBD patients or healthy control people, respectively. (**b**) Five images were randomly captured from each IHC section. Images were analysed using Image-pro Plus 5.0, and the integrated optical densities of TGF-β1 and p-ERK were statistically analysed. Data are presented as the mean±s.d. (*n*=21 in Ctrl group; *n*=23 in patient group). *P* values were generated by unpaired Student’s *t*-test using GraphPad Prism 5 (***P<0.001). (**c**) The correlation between expression of TGF-β1 and p-ERK in intestine tissues was analysed by Spearman correlation analysis using GraphPad Prism 5 (*n*=44).

We measured, using IHC, TGF-β1 and p-ERK levels in intestinal tissues from IBD patients or healthy control people. TGF-β1 and p-ERK levels were significantly higher in intestinal tissues from IBD patients (Fig. 8a,b). After analysing the correlation between levels of TGF-β1 and p-ERK in all intestinal tissues, we found that expression of TGF-β1 was positively correlated to the amount of activated ERK (Fig. 8c). These findings suggest that TGF-β1 expression is upregulated in IBD patients probably in an ERK-dependent manner.

### A33^+^ Li-EVs are bound in the gastrointestinal tract by EpCAM

**Figure 9:**
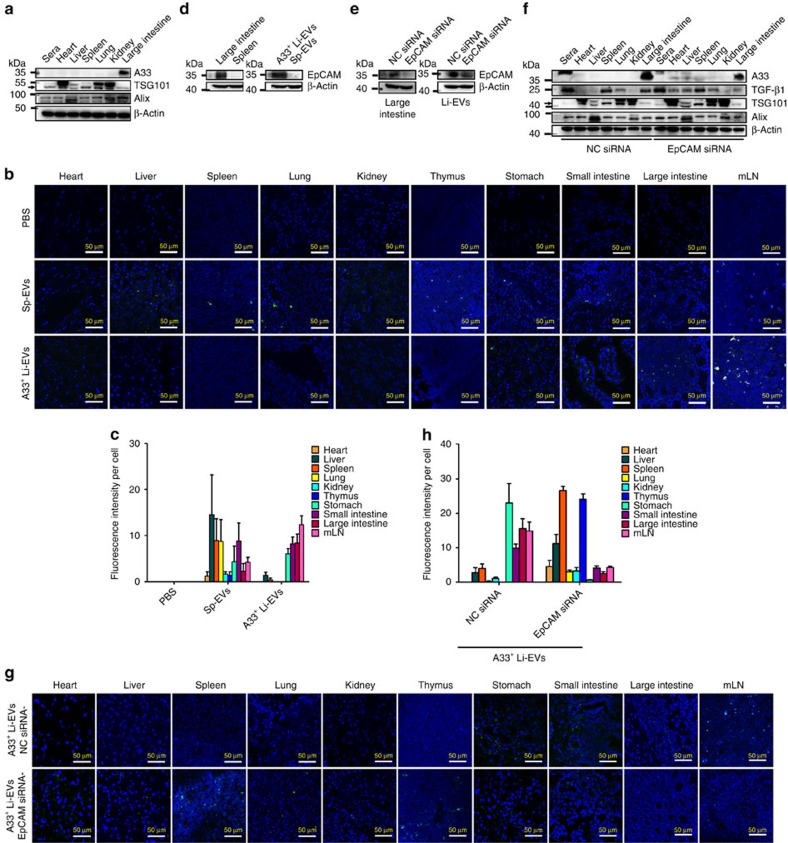
A33^+^ Li-EVs tend to localize in the gastrointestinal tract. (**a**) EVs from sera, heart, liver, spleen, lung, kidney and large intestine were isolated. A33, TSG101, Alix and β-Actin in these EVs were measured using western blot analysis. (**b**) Each mouse received one intravenous injection of 100 μg CFSE-labelled A33^+^ Li-EVs, mice were killed 4 h later and distribution of CFSE-labelled A33^+^ Li-EVs in the indicated organs was detected with an immunofluorescence assay. (**c**) Quantification of the mean green fluorescence intensity per cell (*n*=3). (**d**) EpCAM protein levels in spleen lysates, large intestine lysates, Sp-EVs and A33^+^ Li-EVs were measured using western blot analysis. (**e**–**g**) Mice were intrarectally injected with 20 μg cholesterol-conjugated EpCAM or NC siRNA for three consecutive days. Twenty-four hours after the last injection, mice were killed and A33^+^ Li-EVs were isolated. EpCAM protein levels in lysates of large intestine, and A33^+^ Li-EVs were measured using western blot analysis (**e**). EVs from sera, heart, liver, spleen, lung, kidney and large intestine were isolated. A33, TGF-β1, Alix, TSG101 and β-Actin in these EVs were detected using western blot analysis (**f**). Each mouse received one intravenous injection of 100 μg CFSE-labelled EpCAM siRNA-A33^+^ Li-EVs or NC siRNA-A33^+^ Li-EVs, mice were killed 4 h later and distribution of CFSE-labelled EVs in the indicated organs was detected with an immunofluorescence assay (**g**). (**h**) Quantification of the mean green fluorescence intensity per cell (*n*=3). Data are representative of two independent experiments.

Because Sp-EVs contained high levels of TGF-β1, but showed no protective effect on IBD. We studied whether that result was due to different distributions A33^+^ Li-EVs and Sp-EVs *in vivo*. We isolated EVs from sera, heart, liver, spleen, lung and kidney, and then detected the level of A33 protein in these EVs. We confirmed that all the EVs were positive for TSG101 and Alix and found that, except for A33^+^ Li-EVs, other types of EVs were negative for A33 (Fig. 9a). This result suggests that A33^+^ Li-EVs appear unlikely to traffic to other organs and peripheral blood. To further confirm this, we intravenously transferred CFSE-labelled Sp-EVs or A33^+^ Li-EVs and evaluated the distribution of these EVs *in vivo*. Extensive distribution of exogenic Sp-EVs in many organs were observed; however, exogenic A33^+^ Li-EVs tended to traffic to the gastrointestinal organs including the stomach, small intestine, large intestine and mLN (Fig. 9b,c).

To determine which molecules are responsible for the selective location of A33^+^ Li-EVs, we performed reverse-phase nanospray liquid chromatography-tandem mass spectrometry analysis and found that both Sp-EVs and A33^+^ Li-EVs contained a variety of adhesion molecules (Supplementary Table 2). Among the adhesion molecules found in A33^+^ Li-EVs, but not Sp-EVs, we were interested in EpCAM. EpCAM may cause a physical homophilic interaction molecule between IECs and intraepithelial lymphocytes in the mucosal epithelium^27^. Bioinformatics data (http://www.genecards.org) also indicated high expression levels of EpCAM in the gastrointestinal tract (Supplementary Fig. 7a). We confirmed that large intestine and A33^+^ Li-EVs contained high levels of EpCAM, but spleen and Sp-EVs did not (Fig. 9d). To further determine whether EpCAM and A33 were in the same Li-EV subset, we captured Li-EVs using A33 antibody-coated latex beads, and detected EpCAM using fluorescence activated cell sorting (FACS). EpCAM was detected on Li-EVs captured by A33 antibody-coated latex beads and *vice versa* (Supplementary Fig. 7b). In addition, Li-EVs captured by CD63 antibody-coated latex beads showed both A33 and EpCAM staining on Li-EVs (Supplementary Fig. 7c). CD9 staining indicated the successful capture of Li-EVs (Supplementary Fig. 7b,c). We also found that MC38 cells and MC38-EVs contained very low levels of EpCAM compared with the large intestine or A33^+^ Li-EVs (Supplementary Fig. 7d). After intravenous transfer *in vivo*, MC38-EVs showed extensive distribution in many organs corresponding to their absence of EpCAM (Supplementary Fig. 7e).

To further confirm the effect of EpCAM on A33^+^ Li-EVs location, we knocked down EpCAM expression in the large intestine by intrarectal injection with cholesterol-conjugated EpCAM siRNA. Intrarectal injection with cholesterol-conjugated EpCAM siRNA effectively decreased the mRNA and protein levels of EpCAM in the large intestine (Supplementary Fig. 7f and Fig. 9e). The protein level of EpCAM was also decreased in A33^+^ Li-EVs (Fig. 9e). EpCAM knockdown did not affect TSG101 and Alix protein levels in EVs from sera, heart, liver, spleen, lung, kidney and colon. However, A33 protein levels were found in the EVs from the heart, liver, spleen and lung of mice that received EpCAM, but not NC siRNA treatment (Fig. 9f). Along with the increase of A33^+^ EVs in other organs, that in Li-EVs markedly decreased (Fig. 9f). Consistent with the A33 results, TGF-β1 protein levels were found in EVs from the heart and liver and decreased in Li-EVs as well (Fig. 9f). Because of high baseline levels of TGF-β1 themselves, we did not find increased TGF-β1 protein levels in EVs from the spleen and lung (Fig. 9f). Furthermore, after transferring exogenic A33^+^ Li-EVs from mice with EpCAM (EpCAM siRNA-A33^+^ Li-EVs) or NC siRNA (NC siRNA-A33^+^ Li-EVs) treatment *in vivo*, we found that NC siRNA-A33^+^ Li-EVs were still gastrointestinal organ-specific distributions. However, EpCAM siRNA-A33^+^ Li-EV showed an extensive distribution *in vivo*. In addition, the distribution of EpCAM siRNA-A33^+^ Li-EVs in the gastrointestinal tract obviously decreased (Fig. 9g,h). Together, these results indicate that A33^+^ Li-EVs tend to be bound in the gastrointestinal tract requiring the activity of EpCAM.

### EpCAM affects the protective effect of A33^+^ Li-EVs on IBD

**Figure 10:**
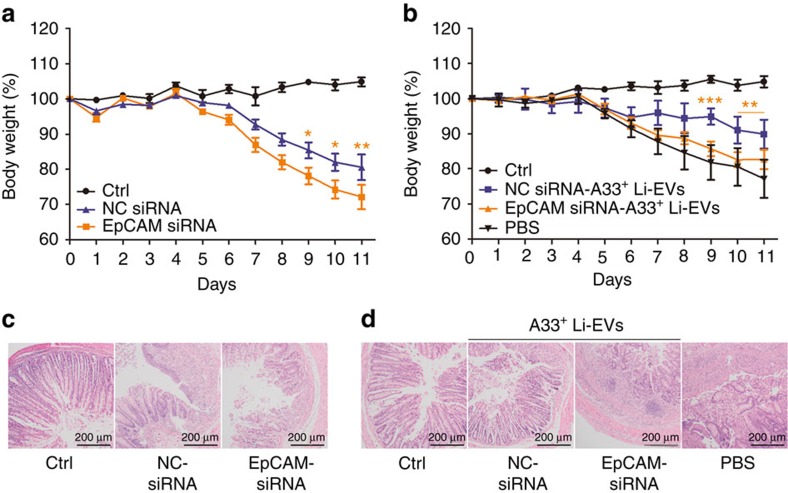
EpCAM effects on the prevention of murine IBD by A33^+^ Li-EVs. (**a**) Mice were fed with 2% DSS solution on day 0 and received intrarectal injection with 20 μg cholesterol-conjugated EpCAM or NC siRNA every other day on days 0–11. The body weights of mice were measured daily. (**b**) Mice with 2% DSS drinking were treated with 100 μg EpCAM siRNA-A33^+^ Li-EVs or NC siRNA-A33^+^ Li-EVs on days −2 and 2. The body weights of mice were measured daily. (**c**,**d**) Histological appearance of colon from mice treated as described in **a**,**b** on days 11. Representative colonic sections stained with H&E. Ctrl group, mice that received normal drinking water; PBS group, mice with drinking water containing 2% DSS and intravenously treated with PBS on days −2 and 2. Data are presented as the mean±s.d. from one of the two independent experiments (*n*=5 per group). *P* values were generated by two-way ANOVA, followed by Newman–Keuls multiple comparison test using GraphPad Prism 5 (*P<0.05, **P<0.01, ***P<0.001), EpCAM siRNA versus NC siRNA in **a** and EpCAM siRNA-A33^+^ Li-EVs versus NC siRNA-A33^+^ Li-EVs in **b**.

To determine whether EpCAM-mediated intestine-specific residence of A33^+^ Li-EVs was responsible for the maintenance of intestinal tract immune balance, after introducing DSS-induced IBD in mice, we found that EpCAM siRNA-treated mice had more severe IBD than did NC siRNA-treated mice (Fig. 10a). Transfer of EpCAM siRNA-A33^+^ Li-EVs or NC siRNA-A33^+^ Li-EVs into IBD mice resulted in a significant NC siRNA-A33^+^ Li-EV-mediated protective effect on murine weight loss. The effect of EpCAM siRNA-A33^+^ Li-EVs was almost totally abolished (Fig. 10b). Histological analysis of colonic damage indicated a similar tendency (Fig. 10c,d). These results indicate that intestine-specific residence of A33^+^ Li-EVs via EpCAM is necessary for their protective effect on murine IBD.

## Discussion

A33^+^ Li-EVs were found to be effective in decreasing DSS-induced murine colonic inflammation. IL-10 and IL-22 are anti-inflammatory cytokines that play protective functions in the development of IBD^28^. Consistent with previous publications, levels of IL-10 and IL-22 increased in DSS-induced colitis^29^,^30^. A33^+^ Li-EVs inhibited, not induced, the production of IL-10 and IL-22. This suggested that the protective effects of A33^+^ Li-EVs on IBD were not IL-10- and IL-22-dependent. IL-10 and IL-22 were induced by the inflammatory microenvironment. After A33^+^ Li-EV treatment, the colonic inflammation was decreased, which probably led to decreased induction of IL-10 and IL-22. Therefore, the decrease in IL-10 and IL-22 may also reflect decreased inflammation in A33^+^ Li-EV-treated mice.

Under normal conditions, the exposure of antigens to DCs is strictly controlled through intestinal barrier function^31^,^32^. After disruption of the intestinal barrier, DCs are exposed to massive amounts of foreign antigens causing excessive activation and subsequent initiation of an inappropriate immune response^9^,^33^. A33^+^ Li-EVs has been shown to inhibit DC activation and antigen-presenting ability, which could explain the DC dependence of their protective effect on IBD. DCs are involved in the development of IBD^22^. However, depletion of DCs in the current study did not increase the severity of IBD. There are different DC subsets possessing distinct effects on the severity of colitis in animal models. CD103^+^ DCs can protect mice from Crohn’s-like ileitis^34^. However, E-cadherin-expressing DCs increase colonic pathology in DSS colitis^35^. Non-selective depletion of protective and pathogenic DCs in CD11c-DTR mice by DT may have been the reason for the lack of difference in IBD severity after DC depletion.

In IBD, it may be that the colon initiates a compensatory mechanism by producing higher TGF-β1 levels, and secreting Li-EVs containing higher levels of TGF-β1 to decrease the severity of IBD. Although we found more severe IBD in spiroepoxide-treated mice, we did not find increased TGF-β1 levels in colon tissues of these mice. There probably is a threshold that even if with more severe IBD, the TGF-β1 level in colon tissues cannot increase further. Alternatively, the severity of IBD induced by spiroepoxide is insufficient to trigger upregulation of TGF-β1 levels.

ERK plays a central role in the upregulation of TGF-β1 in A33^+^ Li-EVs. We could not detect the increased TGF-β1 in the A33^+^ Li-EVs of IBD mice and found an increased IBD symptom after blocking of ERK signalling. These results indicated that in the absence of the compensatory mechanism, IBD was more severe. Moreover, inhibition of ERK activation in healthy mice could also decrease the TGF-β1 in A33^+^ Li-EVs, suggesting the necessity of ERK in maintaining TGF-β1 in A33^+^ Li-EVs under normal physiological conditions. In summary, ERK-dependent maintenance or increase in TGF-β1 levels in A33^+^ Li-EVs or IBD-A33^+^ Li-EVs is important for the immune homeostasis of the intestinal tract. The data suggest that ERK agonists may be effective for IBD treatment.

According to western blot analyses, A33^+^ EVs were not detected in other organs and peripheral blood. In addition, transferred exogenous A33^+^ Li-EVs tended to be located in the gastrointestinal tract. These results suggest that A33^+^ Li-EVs were confined to the intestine. Immunofluorescent analysis of distribution of exogenous A33^+^ Li-EVs also revealed that more A33^+^ Li-EVs were found in mLN than in the spleen, which was consistent with the result that transfer of A33^+^ Li-EVs resulting in a higher percentage of CD4^+^Foxp3^+^ Tregs in mLN than in the spleen. Although Sp-EVs contained higher levels of TGF-β1 than A33^+^ Li-EVs, they had no protective effect to murine IBD. Unlike A33^+^ Li-EVs, Sp-EVs were non-selectively distributed *in vivo,* which may have led to dilution of TGF-β1 in Sp-EVs and ineffectiveness on immunosuppression. These results suggest that organotropism plays a critical role in determining EV functions in the corresponding organ.

In colorectal cancer cells, it has been demonstrated that EpCAM- or A33-positive exosomes are apical or basolateral exosomes, respectively^36^,^37^. It is difficult to discriminate which subsets of physiological A33^+^ Li-EVs are apical or basolateral EVs from IECs. According to our results, Li-EVs contained EpCAM and A33. Unlike MC38 tumour cells and EVs from those cells, in which little EpCAM could be detected, EpCAM was rich in intestinal lysates and A33^+^ Li-EVs. In addition, CD63 was present in both EpCAM- and A33-positive Li-EVs. However, CD63 was only found in apical EVs from colorectal cancer cells^36^. Therefore, EVs from tumour cell lines do not reflect physiological conditions.

After knockdown of EpCAM, leakage of A33^+^ EVs to other organs could be detected and gastrointestinal tract specificity of exogenous A33^+^ Li-EVs disappeared. These results suggest that EpCAM determines the adhesion of A33^+^ EVs in the gastrointestinal tract. Because mice with EpCAM knockdown were more susceptible to DSS-induced IBD and EpCAM siRNA-A33^+^ Li-EVs were ineffective in delaying DSS-induced murine IBD. In addition, the low levels of EpCAM in MC38 cells and non-selective distribution of transferring MC38-EVs *in vivo* suggest that EpCAM may be involved in detachment from the colon epithelium and subsequent remote metastasis of colon cancer cells.

In conclusion, we have demonstrated that IECs can produce EVs containing high levels of TGF-β1. These EVs inhibited CD4^+^ T-cell proliferation *in vitro*. Alteration of the quantity of these EVs affected the severity of DSS-induced IBD in mice. The levels of TGF-β1 in A33^+^ Li-EVs of IBD mice increased in an ERK-dependent manner. In addition, EVs released from DSS-induced IBD mice induced Tregs and regulated DC function. These results reveal a novel mechanism by which intestinal tract immunotolerance is regulated, and suggest that increased EV release from IECs or activation of ERK signalling in IECs may be a promising strategy to restore immune balance in the intestinal tract in IBD.

## Methods

### Human samples

Human paraffin-embedded colon sections from IBD patients were obtained from the Second Affiliated Hospital, Zhejiang University. Normal control colon sections consisting of healthy tissue from the resection edges of tumour biopsies that appeared to be healthy at the histological level were obtained from the Zhejiang Cancer Hospital. The collection of human samples was approved by the local Ethical Committee and the Review Board of the Second Affiliated Hospital, Zhejiang University (number 2015-025) and the Zhejiang Cancer Hospital (number IRB-2015-186). All the patients were informed of the usage of their tissue samples and content forms were obtained. The basic information from all of the patients, including age, sex and colitis location, is summarized in Supplementary Table 3.

### Mice and cell lines

Female C57BL/6J (6–8-week old) mice were purchased from Joint Ventures Sipper BK Experimental Animal Co. (Shanghai, China). *Smad3+/−* and DTR-CD11c mice were purchased from Jackson Laboratory (Farmington, CT, USA). Female mice, 6–8-week old, were used in our experiments. Mice were housed in a specific pathogen-free facility, and the experimental protocols were approved by the Animal Care and Use Committee of School of Medicine, Zhejiang University (Hangzhou, China). MC38 was obtained from American Type Culture Collection (Manassas, VA, USA).

### Exosome isolation and characterization

Intestinal tissues were detached and ground in PBS, and enzymatically digested for 2 h with 1 mg ml^−1^ type II collagenase (Sigma-Aldrich, St Louis, MO, USA; for EV isolation) or mechanically homogenized (for total vesicle isolation). Heart, liver, spleen, lung and kidney were ground in PBS. All the tissue fragments containing supernatants were centrifuged at 300*g* for 10 min. The supernatants were collected and filtered using a 0.22-μm filter and then ultracentrifuged at 100,000*g* for 1 h. The EV pellets were washed in 30 ml of sterile PBS and centrifuged at 100,000*g* for an additional 1 h. The final pellets were resuspended in PBS. Exosomes from murine serum were isolated using ExoQuick EV precipitation solution (System Biosciences Inc., Mountain View, CA, USA) according to the manufacturer’s instructions. The amount of exosomal proteins recovered was measured by a BCA assay (Thermo Fisher, Waltham, MA, USA). The morphology of the isolated EVs was determined using electron microscopy. Exosome pellets were fixed in 4% paraformaldehyde at 4 °C for 1 h. Then, the pellets were loaded on electron microscopy grids coated with formvar carbon, contrasted and embedded in a mixture of uranyl acetate and methylcellulose. Sections were observed with a Philips Tecnai-10 transmission electron microscope operating at 80 kV (Phillips Electronic Instruments, Mahwah, NJ, USA)^38^. To isolate EVs with different density, EVs in 15 ml PBS were loaded on a continuous sucrose gradient consisting of eight fractions with sucrose concentration ranging from 1.07 to 1.21 g ml^−1^ and centrifuged for 2 h at 100,000*g* (ref. 39). Fractions of the gradient (1 ml each) were diluted in 2 ml PBS and centrifuged for 1 h at 100,000*g.*

### Western blot and FACS analysis of EVs

For western blot detection, a total of 30 μg EVs or crude proteins extracted from cell lysates was separated by 12% SDS–PAGE and transferred on polyvinylidene difluoride membrane (Millipore, Billerica, MA, USA). Membrane was blocked with 5% BSA in TBST and then incubated with corresponding primary antibodies overnight at 4 °C. After incubating with horseradish peroxidase-coupled secondary antibodies for 1 h, the membranes were scanned using Tanon 4500 (Shanghai, China), according to the manufacturer’s instructions. For FACS analysis, 20 μg EVs were incubated with 5 μl 4-μm-diameter aldehyde/sulfate latex beads (Invitrogen, New York, NY, USA) for 15 min at room temperature in PBS, with 20 μl final volume. The mixture was then transferred to 1 ml PBS with gentle shaking for 1 h. After centrifugation, the pellet was blocked by incubation with 20 μl fetal bovine serum for 30 min. EV-coated beads were washed three times in PBS and resuspended in 50 μl PBS. Afterwards, beads were incubated with corresponding fluorescent antibodies for 1 h at room temperature in the dark. Beads were analysed using FACS (Becton Dickinson, Franklin Lakes, NJ, USA)^38^. To capture and detect EVs by antibody-coated latex beads, 5 μl latex beads were mixed with 50 μg corresponding antibodies at 4 °C for 1 h and blocked with fetal bovine serum. The beads were washed and resuspended in PBS. Subsequently, the beads were incubated with corresponding detection antibodies and characterized by FACS^40^. To detect TGF-β1 in MC38 cells or MC38 cell-derived EVs (MC38-EVs), MC38 cells, pretreated with or without 10 μM ERK-specific inhibitor U0126 or JNK-specific inhibitor SP600125 (SelleckBio, Houston, TX, USA) for 30 min, were treated with 1 mg ml^−1^ colon tissue lysates from IBD (IBD lysates) or healthy control (Ctrl lysates) mice for 24 h. MC38 cells were treated with 1 mg ml^−1^ IBD lysates or Ctrl lysates mice for the indicated time. The fluorescence-labelled v against CD4 (GK1.5; 1:100), CD8 (53-6.7; 1:100), CD9 (eBioKMC8; 1:100), CD11c (N418; 1:100), B220 (RA3-6B2; 1:100), F4/80 (BM8; 1:100), MHC-II (NIMR-4; 1:100), EpCAM (G8.8; 1:100) and FasL (MFL3; 1:100) were from eBioscience (San Diego, CA, USA). The fluorescence-labelled antibodies against A33 (orb15687; 1:100) were obtained from Biorbyt (Cambridge, Cambridgeshire, UK). Primary antibodies against HSP70 (3A3) (1:500), CD63 (Y-18; 1:500), TGF-β1 (V; 1:500), β-actin (I-19; 1:500), EpCAM (A-20; 1:500), ERK (MK1; 1:500), JNK (D-2; 1:500), p38 (C-20; 1:500), AKT (C-20; 1:500) and the corresponding phosphorylated antibodies for western blot were from Santa Cruz Biotechnology (Santa Cruz, CA, USA). Primary antibodies against GRP94 (9G10; 1:3,000), Calnexin (AF18; 1:3,000), GM130 (EP892Y; 1:3,000), A33 (EPR4240; 1:3,000) and TSG101 (EPR7130(B); 1:3,000) were from Abcam (Cambridge, MA, USA). Primary antibodies against Alix (12422-1-AP; 1:1,000) were from Proteintech (Rosemont, IL, USA). Full-sized scans of western blots are provided in Supplementary Fig. 8.

### T-cell proliferation assay

Murine splenic CD4^+^ T cells isolated by a CD4^+^ T-cell isolation kit II (Miltenyi Biotec, Bergisch Gladbach, Germany) were labelled with CFSE (Invitrogen), according to the manufacturer’s instructions. The labelled CD4^+^ T cells (1 × 10^6^ ml^−1^) were stimulated with 1 μl anti-CD3/CD28-coated beads (Invitrogen) with or without different doses of EVs. To block the TGF-β1 signal, 0.5 μg ml^−1^ SB525334, a potent and selective inhibitor of TGF-β receptor I (SelleckBio)^41^, or 10 μg ml^−1^ anti-TGF-β1-neutralized antibodies (9016, R&D, Minneapolis, MN, USA) were added at the beginning of stimulation. Three days later, the cells were harvested and analysed using FACS.

### Isolation of Li-EV subsets

To isolate Li-EV subsets, a total of 200 μg Li-EVs from healthy or IBD mice with or without 100 mg kg^−1^ ERK inhibitor CI-1040 (SelleckBio) treatment were mixed with CD11c magnetic beads (Miltenyi Biotec; 1 μl μg^−1^ EVs) and gently shaken overnight at 4 °C. Bead-free supernatant containing CD11c^−^ Li-EVs was collected after precipitating CD11c^+^ Li-EV-coated beads using a magnet. CD11c^+^ and CD11c^−^ Li-EVs were resuspended in 10 ml of PBS and pelleted by centrifugation at 100,000*g* for 1 h.

### Analysis of CD4^+^Foxp3^+^ Tregs

To test the function of A33^+^ Li-EVs in inducing CD4^+^Foxp3^+^ Treg cells *in vitro*, murine-naive CD4^+^ T cells (Miltenyi Biotec; 1 × 10^6^ ml^−1^) were isolated and incubated with 1 μl anti-CD3/CD28-coated beads for 72 h in the presence 30 μg ml^−1^ of A33^+^ Li-EVs with or without 0.5 μg ml^−1^ SB525334. To induce Tregs *in vivo*, mice were injected intravenously with 100 μg A33^+^ Li-EVs in 200 μl PBS via the tail vein. Three days later, the mice were killed, and splenocytes and mLNs were isolated. The percentage of CD4^+^Foxp3^+^ Tregs was analysed using FACS.

### Knockdown of ERK and EpCAM *in vivo*

For knockdown of ERK and EpCAM *in vivo*, cholesterol-conjugated ERK siRNA, EpCAM siRNA or NC siRNA was synthesized (Ribobio Co., Guangzhou, China) for *in vivo* RNA delivery^42^. Target sequences for ERK: 5′-GCUGAAUCACAUCCUGGGUAU-3′ (sense), 5′-AUACCCAGGAUGUGAUUCAGC-3′ (antisense); EpCAM: 5′-CCUACUGGAUCAUCAUUGA-3′ (sense), 5′-UCAAUGAUGAUCCAGUAGG-3′ (antisense); scrambled sequences for ERK and EpCAM: 5′-UUCUCCGAACGUGUCACGU-3′ (sense), 5′-ACGUGACACGUUCGGAGAA-3′ (antisense). Mice were intrarectally injected with 20 μg cholesterol-conjugated siRNA for three consecutive days. Twenty-four hours after the last injection, mice were killed, and A33^+^ Li-EVs were isolated.

### Induction of IBD and A33^+^ Li-EV treatment

WT, *Smad3+/−* or CD11c-DTR mice were randomized into groups with similar average body weights. Acute IBD was induced by giving 2% DSS (MP Biomedicals, Solon, OH, USA; *w*/*v*) with mol wt. 36,000–50,000 in acidified drinking water for 12 days^21^. The day mice started to drink the DSS solution was regarded as day 0. For inhibition of ERK signal, mice received intrarectal injections of 20 μg cholesterol-conjugated ERK siRNA every other day on days 0–11, or CI-1040 (100 mg kg^−1^) was intravenously injected into mice on days 3–11. For protection of IBD, mice were intravenously injected with A33^+^ Li-EVs, IBD-A33^+^ Li-EVs (A33^+^ Li-EVs of IBD mice) or A33^+^ Li-EVs of CI-1040-treated IBD mice (IBD-A33^+^ Li-EVs/CI-1040; 100 μg EVs per mouse per injection) on days −2 and 2. Two control groups of mice were established; one was fed with DSS only and the other with normal water only. In some experiments, CD25^+^ T cells were depleted by intraperitoneal injection of anti-mouse CD25-neutralized monoclonal (PC61.5.3, Abcam; 200 μg per mouse in 200 μl PBS) on days −8, −6 and −4. In some experiments, CD11c^+^ DCs in the CD11c-DTR mouse were depleted by intraperitoneal injection of DT (Sigma-Aldrich; 4 μg kg^−1^ body weight in 200 μl PBS) on days −2 and 2.

### Colon culture and MPO assay

The colons were incised longitudinally and washed four times in HBSS supplemented with penicillin and streptomycin. One-centimeter-long transverse segments were prepared and cultured in serum-free RPMI 1640 medium supplemented with penicillin and streptomycin, L-glutamine and nonessential amino acids. After 24 h, the supernatants were collected, and production of IL-6, TNF-α, IL-1β, IL-10 and IL-22 was measured using ELISA (eBioscience). For measurement of MPO activity, 50 mg of colon tissue were put in 2 ml microcentrifuge tube containing 1 ml of PBS for 30 s and then centrifuged at 13,500*g* for 5 min. The pellets were resuspended in 1 ml of 50 mM PBS (pH 6.0) containing 0.5% hexadecyltrimethylammonium bromide. After three cycles of freeze–thaw, the samples were sonicated (90 s) and centrifuged at 13,500*g* for 5 min, followed by an incubation in water for 120 min at 60 °C. The samples were centrifuged at 13,500*g* for 5 min and then 10 μl of supernatant was added to 200 μl of 1 mg ml^−1^ of dianisidine dihydrochloride and 5 × 10^−4^ % H_2_O_2_. The change in optical density is measured at 450 nm three times at 30 s intervals using spectrophotometer. Human neutrophil MPO (Sigma) was used as standard. One unit of MPO activity is defined as the amount that degraded 1.0 μmol of peroxide per minute at 25 °C (ref. 43).

### DC function assay

To generate bone marrow-derived DCs (BMDCs), BM mononuclear cells were prepared from mouse tibia and femur suspensions by depletion of red cells, and were cultured at a density of 2 × 10^6^ cells ml^−1^ in RPMI 1640 medium supplemented with 10% fetal bovine serum, 10 ng ml^−1^ recombinant mouse granulocyte–macrophage colony-stimulating factor and 1 ng ml^−1^ IL-4 (R&D). Non-adherent cells were gently washed out after 48 h of culture; the remaining loosely adherent clusters were cultured for another 4 days^21^. Then, BMDCs were collected and 1 × 10^6^ ml^−1^ cells were incubated with 1 μg ml^−1^ LPS or LPS plus 30 μg ml^−1^ A33^+^ Li-EVs for 24 h. The levels of IL-12p70 in the supernatant were measured using ELISA (eBioscience). For mixed lymphocyte reaction, LPS-stimulated BMDCs were collected and 1 × 10^5^ ml^−1^ BMDCs were cocultured with CD4^+^ T cells from BALB/c mice at the ratio of 1:10 for 3 days. Six hours before the end of culture, 20 μl alamar Blue (Invitrogen) was added and the fluorescent intensity was detected using a DTX 880 multimode detector (Beckman Coulter, Palo Alto, CA, USA). For assessment of antigen-presenting ability of DCs *ex vivo*, they were sorted from A33^+^ Li-EV-treated mice on day 11 and mixed lymphocyte reaction was performed as described above.

### Histopathology and IHC

The intestinal tissues dissected from individual groups of patients and mouse were immediately fixed in 10% paraformaldehyde. Samples were subjected to H&E and IHC staining. Images of the sections were captured, and positive areas analysed. The quantity of TGF-β1 and p-ERK present was calculated as the mean density defined as the integrated optical density divided by the actual area.

### Inhibition of EV secretion *in vitro* and *in vivo*

For semiquantitative detection of EVs *in vitro*, anti-mouse CD63 antibodies (Y-18, Santa Cruz Biotechnology) were coupled to 4 μm aldehyde/sulfate latex beads (Invitrogen) by incubating 35 μg of v with 1 × 10^8^ beads, followed by blocking of remaining activated groups with 4% BSA in PBS^44^.

In all, 2 × 10^5^ MC38 tumour cells were seeded per well into 24-well plates in RPMI 1640 with 10% FCS in the presence of 0, 5 and 10 μM spiroepoxide, an inhibitor of neutral sphingomyelinase 2 (Santa Cruz Biotechnology). Twenty-four hours later, these supernatants were collected. Cell culture supernatants were filtered through 0.22-μm filters. Filtrates (100 μl) were incubated with 20,000 anti-CD63-coupled beads overnight at room temperature with gentle shaking. Beads were washed and incubated with phycoerythrin-anti-CD9 (MZ3, BioLegend, San Diego, CA, USA) for 30 min on ice. After washing in 2% BSA in PBS, the beads were analysed using FACS.

For inhibition of EV production *in vivo*, on day 0, 2-month-old mice were intraperitoneally injected with spiroepoxide in 200 μl PBS (2 g kg^−1^ body weight per mouse) or 200 μl of 3.75% DMSO PBS control every 48 h for 12 days (six injections total). Mice were killed 24 h after the final injection.

### Confocal microscopy

Mouse was intravenously injected with 100 μg following CFSE-labelled EVs: A33^+^ Li-EVs from normal mice, A33^+^ Li-EVs from mice with cholesterol-conjugated EpCAM siRNA or with negative control siRNA injection, Sp-EVs, or MC38-EVs. Each kind of EVs were suspended in 200 μl PBS and mouse received individual intravenous injection of 200 μl PBS was served as a control. Four hours later, mice were killed and their heart, liver, spleen, lung, kidney, thymus, stomach, small and large intestines, and mLNs were detached and coated with embedding medium. They were sectioned into ∼8-μm-thick slices and applied on glass slides. After fixation and staining with 4,6-diamidino-2-phenylindole, the slices were washed with PBS and examined under an Olympus FluoView FV1000 confocal microscope and imaged using the Olympus FluoView version 1.4a viewer (Olympus). The green fluorescence intensity and numbers of blue dots in each image were counted using MetaMorph Offline.

### Statistical analysis

Data are presented as the mean±s.d. or s.e.m. Comparisons between two groups were made using Student’s *t*-test; comparisons between multiple groups were made by analysis of variance and Newman–Keuls test; and the Spearman rank-order correlation test was used to examine correlations between the TGF-β1 and p-ERK expression in intestinal tissues from IBD patients and healthy people using GraphPad Prism 5 (San Diego, CA, USA). Statistical significance was determined at *P*<0.05.

### Data availability

The authors declare that all data are available within the Article and its Supplementary Information files, or are available from the author upon request.

## Additional information

**How to cite this article:** Jiang, L. *et al*. EpCAM-dependent extracellular vesicles from intestinal epithelial cells maintain intestinal tract immune balance. *Nat. Commun.* **7**, 13045 doi: 10.1038/ncomms13045 (2016).

## Supplementary Material

Supplementary Information Supplementary Figures 1-8 and Supplementary Tables 1-3
