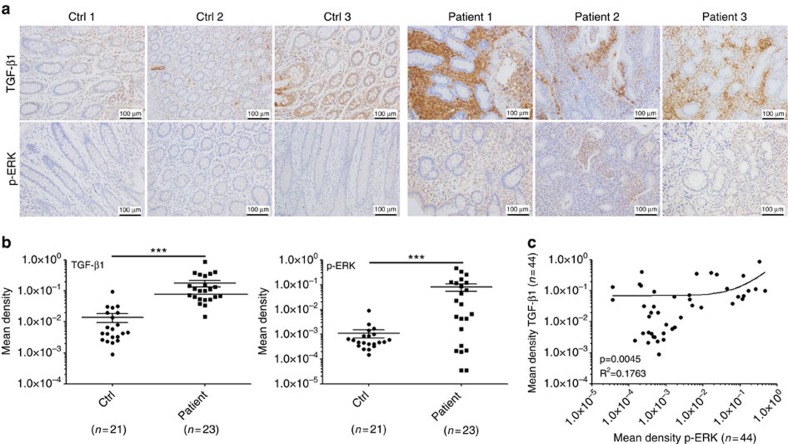# Corrigendum: EpCAM-dependent extracellular vesicles from intestinal epithelial cells maintain intestinal tract immune balance

**DOI:** 10.1038/ncomms16006

**Published:** 2017-06-14

**Authors:** Lingling Jiang, Yingying Shen, Danfeng Guo, Diya Yang, Jiajun Liu, Xuefeng Fei, Yunshan Yang, Buyi Zhang, Zhendong Lin, Fei Yang, Xiaojian Wang, Keyi Wang, Jianli Wang, Zhijian Cai

Nature Communications 7: Article number: 13045; DOI: 10.1038/ncomms13045 (2016); Published: 10 10 2016; Updated: 06 14 2017

This Article contains an error in Fig. 8a, for which we apologize. In Fig. 8a, the control 3 image for p-ERK staining was inadvertently duplicated from the control 1 image of p-ERK staining. The correct version of this figure appears below as Fig. 1.

## Figures and Tables

**Figure 1:**