# Author Correction: EpCAM-dependent extracellular vesicles from intestinal epithelial cells maintain intestinal tract immune balance

**DOI:** 10.1038/s41467-020-17430-y

**Published:** 2020-07-16

**Authors:** Lingling Jiang, Yingying Shen, Danfeng Guo, Diya Yang, Jiajun Liu, Xuefeng Fei, Yunshan Yang, Buyi Zhang, Zhendong Lin, Fei Yang, Xiaojian Wang, Keyi Wang, Jianli Wang, Zhijian Cai

**Affiliations:** 1 Institute of Immunology, Zhejiang University School of Medicine, Hangzhou, 310058 China; 2 Department of Chemotherapy, Zhejiang Cancer Hospital, Hangzhou, 310022 China; 3 Department of Pathology, Second Affiliated Hospital, Zhejiang University School of Medicine, Hangzhou, 310009 China; 4 Department of Gynecology and Obstetrics, Second Affiliated Hospital, Wenzhou Medical University, Wenzhou, 325035 China; 5 Department of Nutrition and Food Hygiene, Zhejiang University School of Public Health, Hangzhou, 310058 China; 6 Chronic Disease Research Institute, Zhejiang University School of Public Health, Hangzhou, 310058 China; 7 Central Laboratory, Nanjing Medical University, Affiliated Hangzhou Hospital (Hangzhou First People’s Hospital), Hangzhou, 310006 China

Correction to: *Nature Communications* 10.1038/ncomms13045, published online 10 October 2016.

The original version of this article contained errors in Fig. 7 and Supplementary Fig. 6. The b-actin bands shown in the right panel of Fig. 7c were duplicated from the b-actin bands in the left panel of Fig. 7e. The incorrect version of the figure is shown below. 

The correct version of Fig. 7c is shown below. 

In Supplementary Fig. 6b, the b-actin band was duplicated from the b-actin band in Fig. 7a. The incorrect version of Supplementary Fig. 6 is shown below. 

The correct version of Supplementary Fig. 6b is shown below. 

In addition, figure legends of Figs. 1, 2, 3, 7 and 9 in the main text and Supplementary Figs. 1, 5, 6, 7 and 8 should have stated that the b-actin gene was used as a sample loading control and was not run on the same blots as the protein of interest.

