# Supplementary material for: EpCAM-dependent extracellular vesicles from intestinal epithelial cells maintain intestinal tract immune balance
Source: Nat Commun. 2016 Oct 10;7:13045. doi: 10.1038/ncomms13045 (PMC5062543; doi:10.1038/ncomms13045)
Supplement: Supplementary Information — Supplementary Figures 1-8 and Supplementary Tables 1-3 [file ncomms13045-s1.pdf]

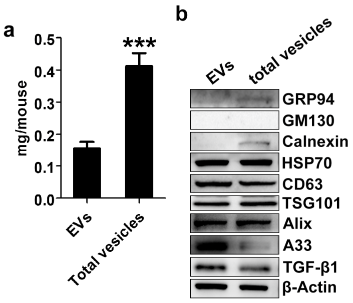

Supplementary Figure 1. Characterization of EVs from intestine. (a, b) Intestinal tissues were ground and enzymatically digested or mechanically homogenized. The EVs or total vesicles were isolated by ultracentrifugation. The quality of vesicles was determined by BCA assay (n=6) **(a)**. Protein components in vesicles were measured by Western blot **(b)**. The data are presented as means ± SEM or represent two independent experiments. *P* values were generated by unpaired student's *t*-test using GraphPad Prism 5 (****P*<0.001).

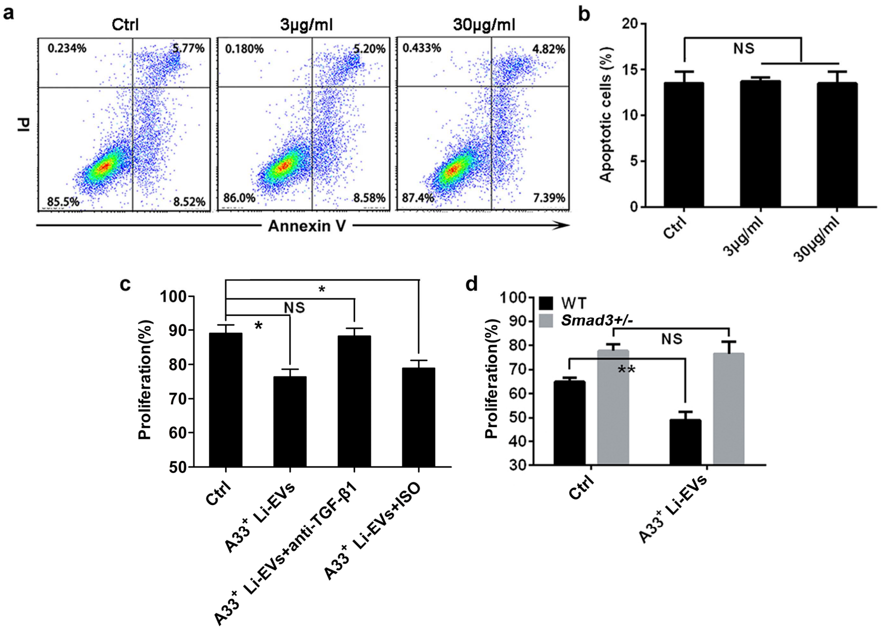

Supplementary Figure 2. Li-EVs from IECs inhibit CD4$^+$ T cell proliferation *in vitro.* (a-c) 1×10^6 ml^{-1} CFSE-labeled CD4$^+$ T cells were seeded into 96-well plate and proliferation of T cells was elicited by 1 μl anti-CD3/CD28-coated beads. The indicated concentrations of Li-EVs were added. After culture for 3 days, apoptosis of CD4$^+$ T cells was measured by annexin V and PI staining **(a)**. The data in **a** were statistically analyzed (n=9) **(b)**. 30 μg ml^{-1} A33$^+$ Li-EVs with anti-TGF-β1-neutralized Abs or isotype control Abs (ISO) were added. After culture for 3 days, proliferation of CD4$^+$ T cells was statistically analyzed (n=6) **(c)**. **(d)** In the presence of 30 μg ml^{-1} A33$^+$ Li-EVs, the proliferation of CD4$^+$ T cells from WT and *Smad3*+/- mice was elicited as described in **a**. Proliferation of CD4$^+$ T cells was statistically analyzed (n=9). The data are present as means ± SEM pooled from three independent experiments. *P* values were generated by one-way ANOVA, followed by Newman-Keuls multiple comparisons test using GraphPad Prism 5 (***P*<0.01, NS, not significant).

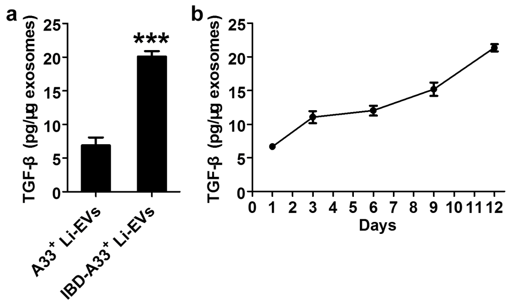

Supplementary Figure 3. Kinetics of TGF-β1 in A33^{+} Li-EVs from mice with IBD. (a, b) EVs were disrupted by three cycles of freeze-thaw. TGF-β1 in A33^{+} Li-EVs and IBD-A33^{+} Li-EVs **(a)** or TGF-β1 in A33^{+} Li-EVs from mice fed with 2% DSS solution for the indicated days **(b)** was detected by ELISA. The data are presented as means ± SEM (n=6) pooled from two independent experiments. *P* values were generated by unpaired student's *t*-test using GraphPad Prism 5 (****P*<0.001).

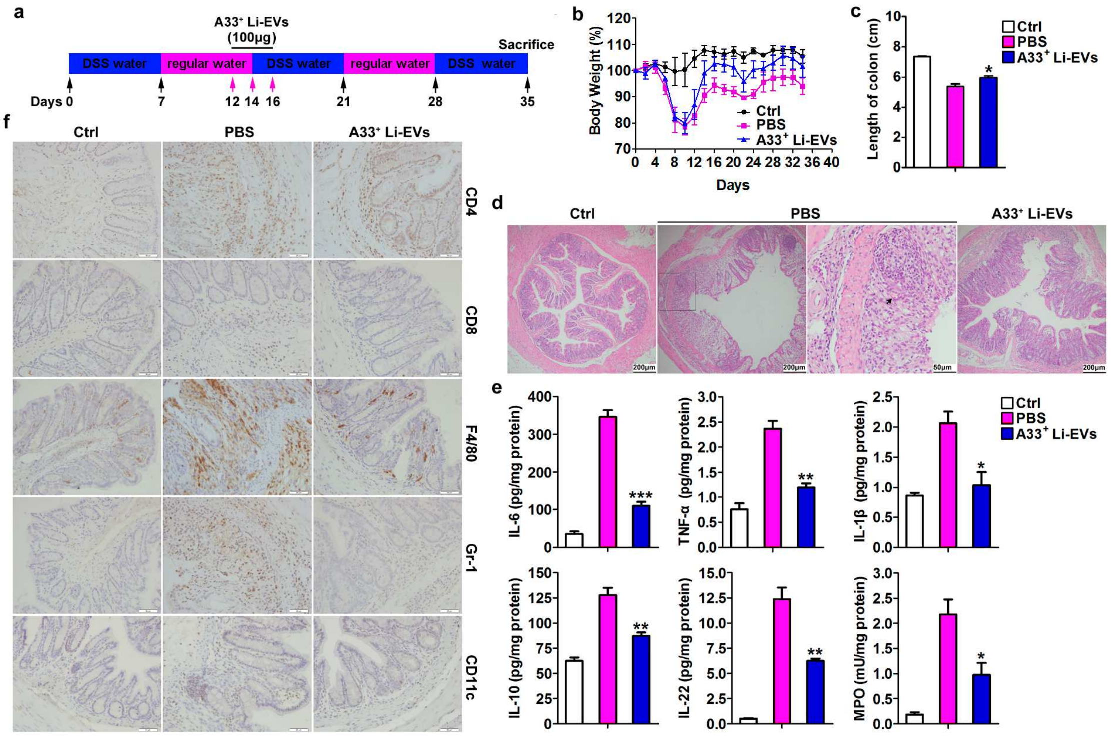

Supplementary Figure 4. Effects of A33$^+$ Li-EVs on murine chronic IBD. (a) Schematic of the DSS protocol for murine chronic IBD induction, and the time points of A33$^+$ Li-EV intravenous injections. **(b)** The body weights were measured every other day. **(c-f)** The mice were sacrificed on day 35. Length of colon was measured **(c)**. Histological appearance of colon tissues after H&E staining. In PBS group, the right image represents a magnification of the dashed region of left image. Arrow indicates a blood vessel **(d)**. IL-6, TNF-α, IL-1β, IL-10 and IL-22 levels and MPO activity in the colon tissues were normalized to the quantity of colon tissue in the samples **(e)**. Expression of CD4, CD8, F4/80, Gr-1 and CD11c was detected by IHC **(f)**. Ctrl group, mice received normal drinking water; PBS group, mice with 2% DSS solution, and intravenously treated with PBS on days 12, 14 and 16. **b,** data are presented as the mean ± SD from one of the two independent experiments (n=5 per group); **c** and **e,** data are shown as mean values ± SEM (n=6) pooled from two independent experiments. *P* values were generated by one-way ANOVA, followed by Newman-Keuls multiple comparisons test using GraphPad Prism 5 (**P*<0.05, ***P*<0.01, ****P*<0.001), versus PBS. Scale bar, 50 μm in **f**.

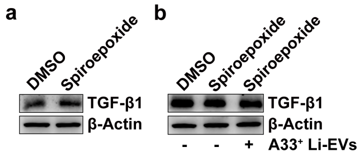

Supplementary Figure 5. TGF-β1 level in colon tissues of spiroepoxide-treated mice. (a) Mice were intraperitoneally injected with spiroepoxide (2 g kg^{-1} body weight) or DMSO control every 48 h for 12 days (6 injections total). The TGF-β1 levels in colon tissues were measured by Western blot. **(b)** Mice were fed with water or 2% DSS solution on day 0 and received intraperitoneal injections of spiroepoxide (2 g kg^{-1} body weight) or DMSO control. In addition to spiroepoxide injection, some mice were also intravenously injected with 100 μg $A33^{+}$ Li-EVs. The TGF-β1 levels in colon tissues were measured by Western blot. Data represent two independent experiments.

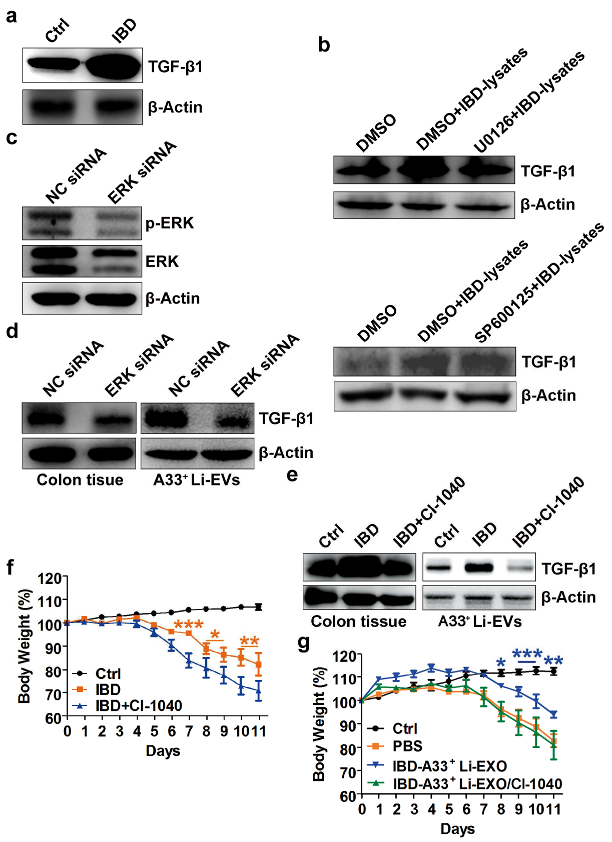

Supplementary Figure 6. ERK mediates increased TGF-β1 levels in IBD-A33$^+$ Li-EVs. (a) TGF-β1 levels in colon tissue lysates from control or IBD mice was measured by Western blot. **(b)** MC38 cells were pre-treated with DMSO, 10 μM ERK specific inhibitor U0126 or JNK specific inhibitor SP600125 for 30 min and then treated with 1 mg ml^{-1} IBD-lysates for 24 h. TGF-β1 levels in MC38 cells was measured by Western blot. **(c, d)** Mice were intrarectally injected with 20 μg cholesterol-conjugated ERK or NC siRNA for three consecutive days. Twenty-four hours after the last injection, mice were sacrificed. Total ERK and p-ERK protein levels in large intestinal lysates were measured by Western blot **(c)**. A33$^+$ Li-EVs were isolated and TGF-β1 protein levels in large intestinal lysates and A33$^+$ Li-.EVs were measured by Western blot **(d)**. **(e-g)** Mice were fed with 2% DSS solution on day 0. Each mouse was intraperitoneally injected with CI-1040 (100 mg kg^{-1}) every day on days 3-11. TGF-β1 levels in colon tissues and A33$^+$ Li-EVs was measured by Western blot **(e)**. The

body weights of mice were measured daily **(f)**. Mice were treated with 100 μg A33$^+$ Li-EVs and IBD-A33$^+$ Li-EVs/CI-1040 on days -2 and 2. The body weights were measured daily **(g)**. Ctrl group, mice received normal drinking water; PBS group, mice with drinking water containing 2% DSS, and intravenously treated with PBS on days -2 and 2. **a-e**, data represent three independent experiments; **f and g**, data are presented as the mean ± SD from one of the two independent experiments (n=5 per group). *P* values were generated by two-way ANOVA, followed by Newman-Keuls multiple comparisons test using GraphPad Prism 5 (**P*<0.05, ***P*<0.01, ****P*<0.001), IBD + DMSO versus IBD + CI-1040 in **f**; A33$^+$ Li-EVs versus IBD-A33$^+$ Li-EVs/CI-1040 in **g**. IBD-A33$^+$ Li-EVs/CI-1040, A33$^+$ Li-EVs of CI-1040 treated IBD mice.

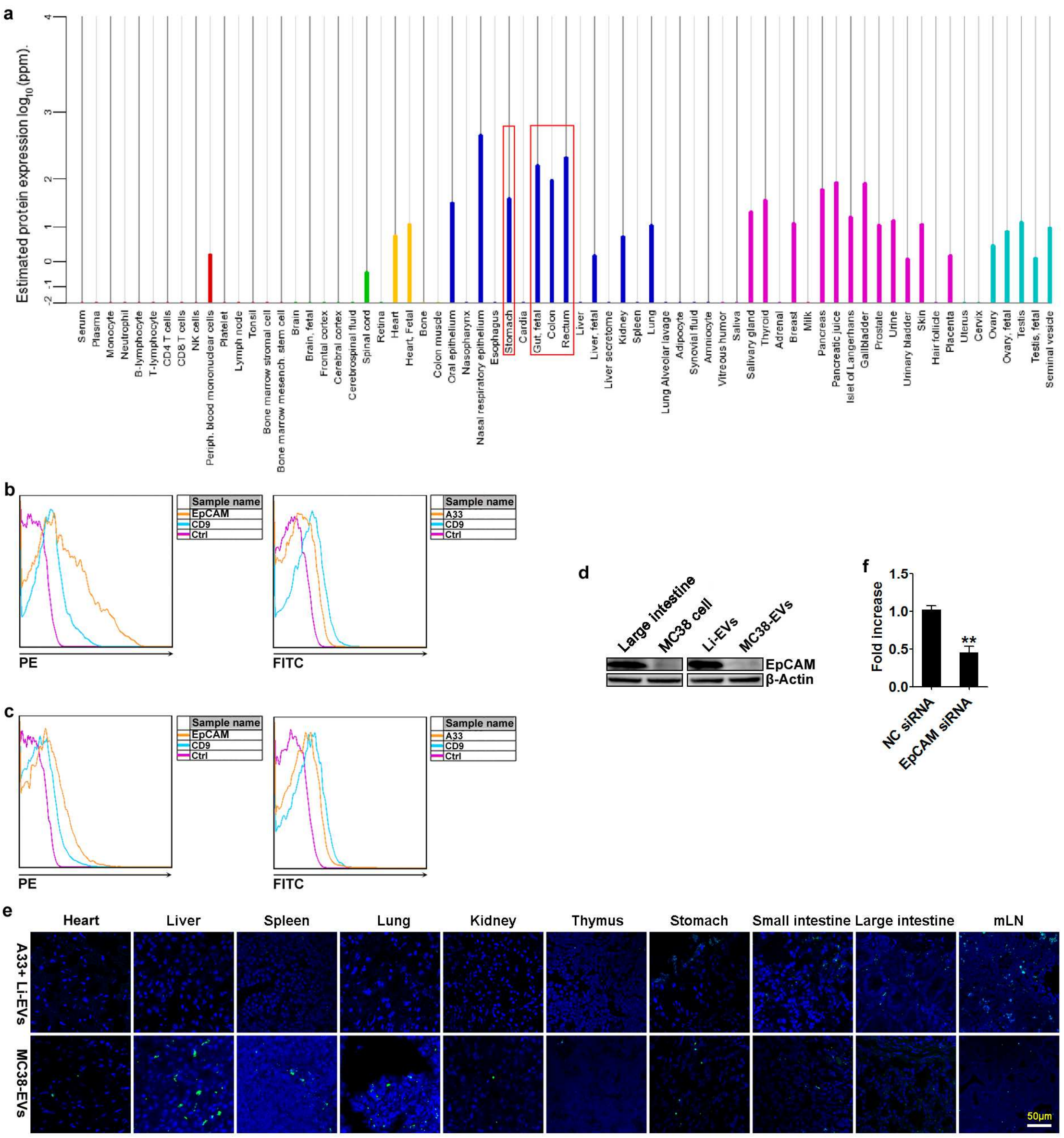

Supplementary Figure 7. EpCAM mediates A33^{+} Li-EV adhesion of gastrointestinal tract. (a) Graph of EpCAM protein level in various cells, organs and body fluids from genecards website. EpCAM protein levels in gastrointestinal tract are marked by red box. **(b)** EpCAM was detected by FACS on Li-EVs captured by A33 Ab-coated latex beads (left), A33 was detected by FACS on Li-EVs captured by EpCAM Ab-coated latex beads (right). **(c)** EpCAM (left) and A33 (right) were detected by FACS on Li-EVs captured by CD63 Ab-coated latex beads. **(d)** EpCAM in MC38 cells, MC38-EVs, large intestine, and A33^{+} Li-EVs levels were measured by Western blot. **(e)** Each mouse received one

intravenous injection of 100 μg CFSE-labeled MC38-EVs or A33^{+} Li-EVs. Mice were sacrificed 4 h later, and the distribution of CFSE-labeled EVs in the indicated organs was determined by immunofluorescence assays. **(f)** Mice were intrarectally injected with 20 μg cholesterol-conjugated EpCAM or NC siRNA for three consecutive days. Twenty-four hours after the last injection, mice were sacrificed and the EpCAM mRNA level in large intestinal lysates was detected by real-time PCR. Data represent two independent experiments or are presented as the mean ± SEM from one of the two independent experiments (n=6). *P* values were generated by unpaired student's *t*-test using GraphPad Prism 5 (***P*<0.01).

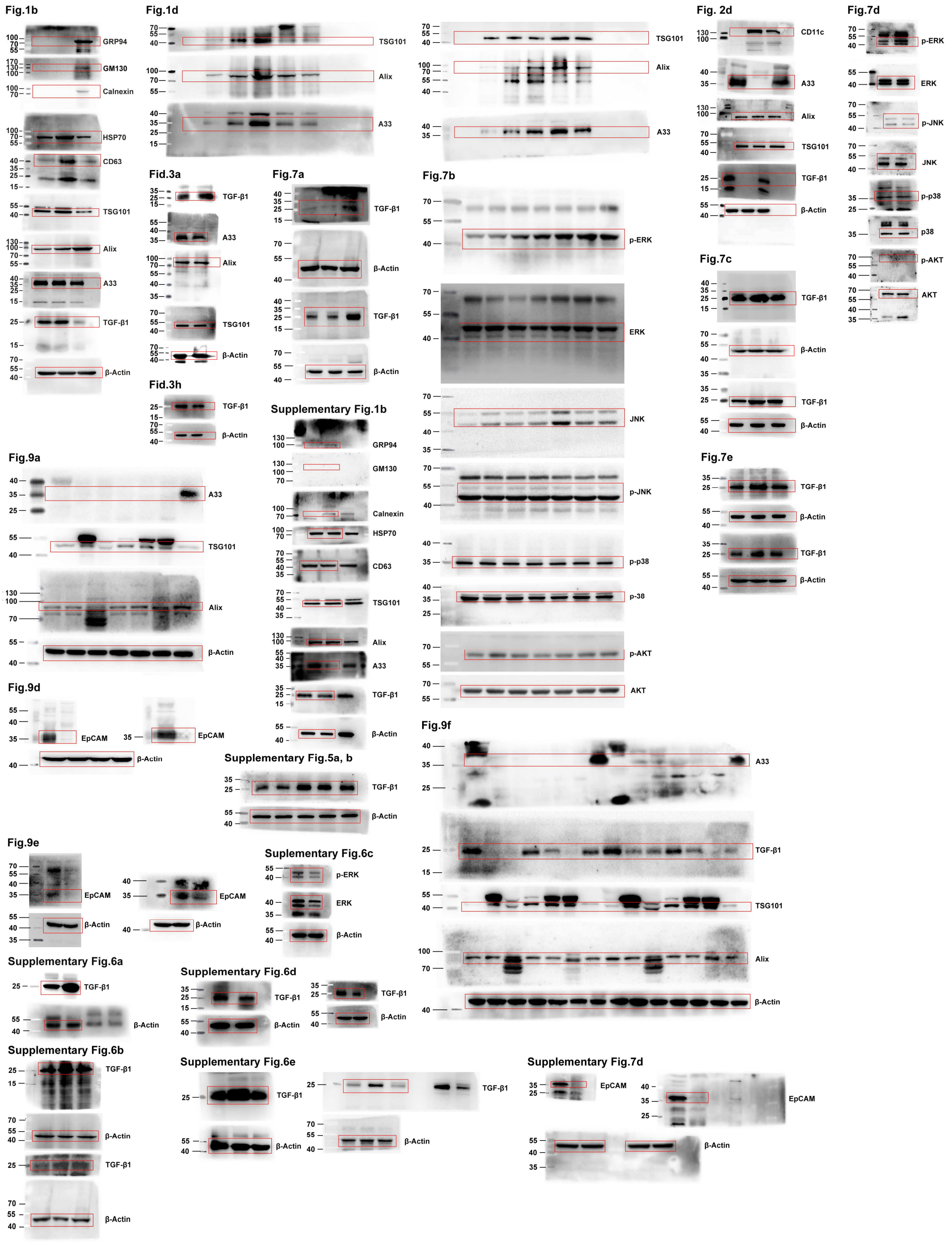

Supplementary Figure 8. Uncropped images of blots presented in the main paper. Molecular weight markers are indicated in kDa.

Supplementary Table 1. Size distribution of intestinal EVs was analyzed by particle size analyzer (BIC, Austin, TX, USA).

	Size (nm)	Polydispersity	Z-potential (mV)
**Si-EVs**	75.76 ± 10.87	0.154 ± 0.001	- 22.22 ± 0.92
**Li-EVs**	73.47 ± 5.92	0.166 ± 0.007	- 22.33 ± 0.62
**IBD-A33^{+} Li-EVs**	74.13 ± 11.5	0.162 ± 0.004	- 22.26 ± 0.35
**CD11c^{+} Li-EVs**	80.90 ± 12.33	0.171 ± 0.006	- 19.43 ± 0.04
**CD11c^{-} Li-EVs**	71.83 ± 10.33	0.165 ± 0.004	- 20.70 ± 0.85

Data denoted means ± SEM from one of the three independent experiments.

-

Supplementary Table 2. Proteins related to adhesion found in EVs.

**Proteins found in Li-EVs only**	**Functions**
Cadherin-related family member 5	Acts as a calcium-dependent cell adhesion protein.
Catenin beta-1	Involved in the regulation of cell adhesion. Acts as a negative regulator of centrosome cohesion.
Claudin-7	Plays a major role in tight junction-specific obliteration of the intercellular space, through calcium-independent cell-adhesion activity.
Desmoglein-1-beta	Component of intercellular desmosome junctions. Involved in the interaction of plaque proteins and intermediate filaments mediating cell-cell adhesion.
Epithelial cell adhesion molecule	May act as a physical homophilic interaction molecule between intestinal epithelial cells (IECs) and intraepithelial lymphocytes (IELs) at the mucosal epithelium for providing immunological barrier as a first line of defense against mucosal infection.
Ezrin	Cell adhesion molecule binding.
Flotillin-2	May be involved in epidermal cell adhesion and epidermal structure and function.
Galectin-3	Involved in acute inflammatory responses including neutrophil activation and adhesion, chemoattraction of monocytes macrophages, opsonization of apoptotic neutrophils, and activation of mast cells.
Galectin-3-binding protein	Promotes intergrin-mediated cell adhesion.
Integrin alpha-2	Collagen binding involved in cell-matrix adhesion.
Integrin alpha-3	Participate in the adhesion, formation of invadopodia and matrix degradation processes, promoting cell invasion.
Integrin alpha-5	Integrin alpha-5/beta-1 is a receptor for fibronectin and fibrinogen.
Integrin beta-4	Is required for the regulation of keratinocyte polarity and

	motility.
Integrin beta-6	Integrin alpha-V/beta-6 is a receptor for fibronectin and cytotactin.
Leukocyte surface antigen CD47	Has a role in both cell adhesion by acting as an adhesion receptor for THBS1 on platelets, and in the modulation of integrins.
Membrane primary amine oxidase	Cell adhesion protein that participates in lymphocyte recirculation by mediating the binding of lymphocytes to peripheral lymph node vascular endothelial cells in an L-selectin-independent fashion.
Neuroplastin	Probable homophilic and heterophilic cell adhesion molecule involved in long term potentiation at hippocampal excitatory synapses through activation of p38MAPK.
Periostin	Induces cell attachment and spreading and plays a role in cell adhesion. May play a role in extracellular matrix mineralization.
Plakophilin-1	Lamin binding
Protocadherin-16	Calcium-dependent cell-adhesion protein. Mediates functions in neuroprogenitor cell proliferation and differentiation.
Talin-1	Integrin binding.
Vinculin	Actin filament (F-actin)-binding protein involved in cell-matrix adhesion and cell-cell adhesion.

**Proteins found in Sp-EVs only**	**Functions**
CD209 antigen-like protein B	Is a receptor for ICAM3, probably by binding to mannose-like carbohydrates
CD97	Receptor potentially involved in both adhesion and signaling processes early after leukocyte activation.
EGF-like module-containing mucin-like hormone receptor-like 1	Orphan receptor involved in cell adhesion and probably in cell-cell interactions specifically involving cells of the immune system.

Fibronectin	Fibronectins are involved in cell adhesion, cell motility, opsonization, wound healing, and maintenance of cell shape healing, and maintenance of cell shape.
Integrin alpha-6	Integrin alpha-6/beta-1 is a receptor for laminin on platelets. Integrin alpha-6/beta-4 is a receptor for laminin in epithelial cells and it plays a critical structural role in the hemidesmosome.
Integrin alpha-D	Integrin alpha-D/beta-2 is a receptor for ICAM3 and VCAM1.
Integrin alpha-Iib	Integrin alpha-IIb/beta-3 is a receptor for fibronectin, fibrinogen, plasminogen, prothrombin, thrombospondin and vitronectin.
Integrin alpha-L	Integrin alpha-L/beta-2 is a receptor for ICAM1, ICAM2, ICAM3 and ICAM4.
Integrin alpha-M	Integrin alpha-M/beta-2 is implicated in various adhesive interactions of monocytes, macrophages and granulocytes as well as in mediating the uptake of complement-coated particles. Integrin alpha-M/beta-2 is a receptor for fibrinogen, factor X and ICAM1.
Integrin alpha-V	The alpha-V (ITGAV) integrins are receptors for vitronectin, cytotactin, fibronectin, fibrinogen, laminin, matrix metalloproteinase-2, osteopontin, osteomodulin, prothrombin, thrombospondin and vWF.
Integrin alpha-X	Integrin alpha-X/beta-2 is a receptor for fibrinogen. It mediates cell-cell interaction during inflammatory responses. It is especially important in monocyte adhesion and chemotaxis
Integrin beta-5	Integrin alpha-V/beta-5 (ITGAV: ITGB5) is a receptor for fibronectin.
Integrin beta-7	Integrin alpha-4/beta-7 (Peyer patches-specific homing receptor LPAM-1) is involved in adhesive interactions of leukocytes. Integrin alpha-4/beta-7 is a receptor for MADCAM1 and VCAM1. Integrin alpha-E/beta-7 is also a receptor for E-cadherin.
Intercellular adhesion molecule 1	ICAM proteins are ligands for the leukocyte adhesion protein

	LFA-1 (integrin alpha-L/beta-2).
Macrosialin	Could play a role in phagocytic activities of tissue macrophages, both in intracellular lysosomal metabolism and extracellular cell-cell and cell-pathogen interactions. Binds to tissue- and organ-specific lectins or selectins, allowing homing of macrophage subsets to particular sites. Rapid recirculation of CD68 from endosomes and lysosomes to the plasma membrane may allow macrophages to crawl over selectin-bearing substrates or other cells
Protein ERGIC-53	Mannose-specific lectin.
Talin-2	As a major component of focal adhesion plaques that links integrin to the actin cytoskeleton, may play an important role in cell adhesion.
Vascular cell adhesion protein 1	Appears to function in leukocyte-endothelial cell adhesion. Interacts with integrin alpha-4/beta-1 (ITGA4/ITGB1) on leukocytes, and mediates both adhesion and signal transduction.

**Proteins found in both EVs**	**Functions**
Integrin beta-1	Regulates cell adhesion and laminin matrix deposition. Involved in promoting endothelial cell motility and angiogenesis.
Integrin alpha-6	Integrin alpha-6/beta-1 is a receptor for laminin on platelets. Integrin alpha-6/beta-4 is a receptor for laminin in epithelial cells and it plays a critical structural role in the hemidesmosome.
Integrin beta-2	Receptor for ICAM1, ICAM2, ICAM3 and ICAM4. Cell adhesion molecule binding.
Integrin beta-3	Following activation integrin alpha-IIB/beta-3 brings about platelet/platelet interaction through binding of soluble fibrinogen. This step leads to rapid platelet aggregation which physically plugs ruptured endothelial surfaces.

Supplementary Table 3. Basic information of normal control and IBD patients.

	**Normal control**	**IBD**
	n=21	n=23
**Age (years)**		
<=30	0	4
30-50	5	5
=>50	16	14
**Gender**		
Male	9	9
Female	12	14
**Location**		
Colon	20	16
Terminal ileum	0	1
Rectum	1	4
Ileocecal	0	2
